# Immunological testing and machine learning in detecting latent tuberculosis among high-risk groups (nature review)

**DOI:** 10.3389/fmed.2025.1710960

**Published:** 2026-01-07

**Authors:** Anna Starshinova, Adilya Sabirova, Igor Kudryavtsev, Artem Rubinstein, Leonid P. Churilov, Ekaterina Belyaeva, Kulpina Anastasia, Raul A. Sharipov, Ravil K. Tukfatullin, Nikolay Nikolenko, Irina Dovgalyuk, Dmitry Kudlay

**Affiliations:** 1 Department of Mathematics and Computer Science, Saint Petersburg State University, Saint Petersburg, Russia; 2 Almazov National Medical Research Center of the Ministry of Health of the Russian Federation, Saint Petersburg, Russia; 3 Medical Department, Bashkir State Medical University, Ufa, Russia; 4 Institute of Experimental Medicine, Saint Petersburg, Russia; 5 Medical Department, Saint Petersburg State University, Saint Petersburg, Russia; 6 The Moscow Research and Clinical Center for Tuberculosis Control of the Moscow Government Department of Health, Moscow, Russia; 7 St. Petersburg Research Institute of Phthisiopulmonology, Saint Petersburg, Russia; 8 Department of Pharmacology, Institute of Pharmacy, I.M. Sechenov First Moscow State Medical University (Sechenov University), Moscow, Russia; 9 Scientific and Technological Development of the Faculty of Bioengineering and Bioinformatics, Faculty of Bioengineering and Bioinformatics, Lomonosov Moscow State University, Moscow, Russia; 10 Laboratory of Personalized Medicine and Molecular Immunology, NRC Institute of Immunology FMBA of Russia, Moscow, Russia

**Keywords:** tuberculosis infection, risk groups, early diagnosis, latent tuberculosis infection, immunological tests, T-cell response, machine learning

## Abstract

**Introduction:**

Tuberculosis infection remains one of the most dangerous and difficult to diagnose diseases. To date, issues related to the early diagnosis of tuberculosis remain unresolved, which is particularly important for its detection in high-risk groups. The detection of latent tuberculosis infection (LTBI) is necessary to control the spread of tuberculosis infection. The diagnosis of LTBI is indirect and based on the detection of an immune response to mycobacterial antigens. Currently, LTBI diagnosis is recommended in high-risk groups. However, diagnosis is difficult and not always straightforward with the use of various immunological tests. The aim of this study is to conduct a systematic review of scientific publications focused on the application of immunological tests and machine learning technologies for the early detection of latent tuberculosis infection in high-risk populations.

**Material and Methods:**

We analyzed articles for the period from 2015 to 2025, published in international databases (Medline, PubMed, Scopus). The keywords we used were “tuberculosis infection,” “risk groups,” “early diagnosis,” “latent tuberculosis infection,” “immunological tests,” “T-cell response,” and “machine learning.” The narrative review was carried out in accordance with the PRISMA protocol (http://www.prisma-statement.org).

**Results:**

A descriptive research method was used to compile the review, followed by systematization of the information and formulation of the main conclusions. The data obtained allow us to assert that the use of a comprehensive approach in the diagnosis of LTBI, namely the simultaneous use of several immunological tests in combination with laboratory and instrumental research methods in the same individuals, can be considered justified.

**Conclusion:**

The creation of a strategy for detecting LTBI in individuals from risk groups can facilitate the detection of infection and play an important role in preventing the development of tuberculosis. The possibility of using machine learning and artificial intelligence will allow the risk of developing active tuberculosis to be determined based on the use of immunological tests.

## 1 Introduction

Tuberculosis infection remains one of the most dangerous and difficult to diagnose diseases. To date, issues surrounding the early diagnosis of tuberculosis remain unresolved, which is particularly important for its detection in high-risk groups. Unfortunately, the COVID-19 pandemic has had an impact on the dynamics of tuberculosis incidence and the spread of the disease among the most vulnerable high-risk patient groups. This risk increases significantly in individuals with latent tuberculosis infection, especially after COVID-19 and in the presence of post-COVID syndrome (1, 2). According to estimates by the World Health Organization (WHO), 10.8 million people worldwide contracted tuberculosis in 2023, which is equivalent to 134 cases per 100,000 population. Between 2021 and 2023, there was a steady increase in the incidence of tuberculosis worldwide: 10.4 million in 2021, 10.7 million in 2022, and 10.8 million in 2023 (3). The increase in global tuberculosis incidence can be partly explained by the COVID-19 pandemic. According to foreign authors, quarantine may lead to the development of active tuberculosis in individuals with latent tuberculosis infection (LTBI) who have not received preventive therapy, for example, those who have recently been in contact with tuberculosis patients or individuals with weakened immune systems (4). As is well known, back in 2022, the Russian Federation (RF) was removed from the list of countries with a high burden of tuberculosis (5). According to the WHO Global Tuberculosis Report, in 2024 the incidence of tuberculosis in Russia in 2023 was 38 per 100,000 population. Over the period 2015-2023, it decreased by 43%. The total number of deaths from tuberculosis over the period 2015-2023 decreased by 58% (3). The incidence of tuberculosis over the previous 13-year period has generally declined in the Russian Federation, reaching 26.5 cases per 100,000 population in 2024, which is almost half the average annual rate (49.4). A total of 38,753 newly diagnosed cases were registered in 2024, which is 9.65% less than in 2023 (6). However, despite this, the total economic cost of combating tuberculosis in 2024 amounted to 129.5 billion roubles. In our opinion, timely detection of LTBI and the prescription of preventive therapy would help to reduce this economic burden (6). In 2019, a systematic review and meta-analysis was published, which concluded that the prevalence of latent tuberculosis infection [LTBI) was 25% (95% CI 19.7–30.0%) and 21.2% (95% CI 17.9–24.4%]. Based on this, it can be assumed that about a quarter of the world’s population has LTBI (7). The WHO recommendations identify categories of the population that should be considered at risk for developing tuberculosis (8). The WHO makes an amendment stating that systematic testing for LTBI is not recommended for patients with diabetes mellitus, alcohol abusers, tobacco smokers, and underweight individuals, unless these individuals have been classified as risk groups. People with these risk factors require closer monitoring and diagnosis (8, 9). The aim of this study is to conduct a systematic review of scientific publications focused on the application of immunological tests and machine learning technologies for the early detection of latent tuberculosis infection in high-risk populations.

## 2 Materials and methods

An analysis was conducted of articles published from 2015 to 2025 in international databases (Medline, PubMed, Scopus, Web of Science, and Google Scholar). The following keywords were used: “tuberculosis infection”, “risk groups”, “early diagnosis,” “LTI” (“latent tuberculosis infection”), “immunological tests,” “machine learning”. A descriptive review was carried out according to the PRISMA protocol.^1^ For compiling the review, a descriptive research method was applied with subsequent systematization of information and formulation of main conclusions. Inclusion criteria: Studies addressing the diagnosis of latent tuberculosis infection (LTBI); Use of immunological tests, evaluation of T-cell responses or implementation of machine learning algorithms. Assessment of the effectiveness of early diagnosis in at-risk groups. Original research articles. Exclusion criteria: Review articles, clinical guidelines, and recommendations. Studies not directly related to the early detection of LTBI. Articles containing incomplete or outdated data.

## 3 Latent tuberculosis infection

LTBI is characterized by the presence of an immune response to *Mycobacterium tuberculosis* infection without clinical signs of active tuberculosis. The lifetime risk of tuberculosis reactivation for a person with documented LTBI is estimated at 5–15%, with most people developing tuberculosis within the first 5 years after initial infection. An important aspect of tuberculosis prevention is screening potential risk groups for LTBI development: these are people living with HIV, contacts, patients receiving immunosuppressive therapy, including those who have undergone organ transplantation, those on haemodialysis, and to a lesser extent, healthcare workers, prisoners, immigrants from countries with a high burden of tuberculosis, etc. (10). According to current data, the persistence of mycobacteria in the human body, which provides a substrate for LTBI, is made possible by mechanisms such as dormancy, drug tolerance, L-transformation, and intercellular communication between bacteria (the quorum sensing phenomenon). In this regard, LTBI is a constant reservoir for the possible development of active tuberculosis (11). The identification of tuberculosis infection [according to Drain et al. (50)] at different heterogeneous stages after initial contact with a patient with tuberculosis: at the stages of pathogen elimination, at the LTBI stage, i.e. when mycobacteria are metabolically inactive, and at the initial preclinical stage. The identification of these conditions undoubtedly allows for the timely implementation of appropriate measures, such as chemoprophylaxis, and reduces the number of cases of manifest tuberculosis. However, there are difficulties, as there is no universal diagnostic method that allows these conditions to be reliably distinguished (12). Currently, MDR-TB is of particular interest due to the spread of its multidrug-resistant forms. According to some estimates, 3 out of 1,000 people worldwide have multidrug-resistant MDR-TB, which may create serious problems for controlling the spread of MDR-TB in the future (13).

## 4 Immunodiagnostic methods

Currently, there is no test for direct detection of LTIs in humans. The diagnosis of LTBI is indirect and based on the detection of an immune response to mycobacterial antigens. The classic method for detecting LTBI is the tuberculin skin test [Mantoux test (TST)] *in vivo*, but its specificity is largely influenced by previous BCG vaccination (9). Extensive experience has been gained in the use of interferon-γ release assays (IGRA). New tests have been developed that retain the basis of IGRA but are performed on automated laboratory analysers. For example, VIDAS TB-IGRA (bioMérieux) has demonstrated high concordance with QuantiFERON-TB Gold Plus and a reduction in the proportion of indeterminate results due to process standardization (14). Similarly, LIAISON^®^ QuantiFERON-TB Gold Plus (DiaSorin/QIAGEN) enables IGRA to be performed in a high-throughput chemiluminescence assay format (14). *In vitro* tests based on the release of γ-interferon by sensitized lymphocytes after stimulation with antigens, as well as the determination of sensitized T lymphocytes by specific ESAT6 and CFP10 antigens (ELISPOT (The USA), QuantiFERON-TB Gold (The UK), QuantiFERON-TB Gold Plus (The UK), WANTAI TB-IGRA (China), TigraTest-TB^®^ (Russia), are widely used in the USA and European countries (15–17). This is due to the high specificity and sensitivity of the tests, but their high cost is a significant limitation to their use.

The relevance of IGRA tests has been proven in numerous studies conducted to date, despite their proven high specificity and sensitivity. One recent study compared the results of VIDAS TB-IGRA (manufactured in France) with the previously established QuantiFERON-TB Gold Plus (manufactured in Germany) to assess their diagnostic effectiveness in the diagnosis of LTBI. The study included 104 patients with tuberculosis, 162 people at high risk and 117 people at low risk of developing tuberculosis. PPA (Positive Percent Agreement) and NPA (Negative Percent Agreement) indicators were used to assess consistency. The study found that VIDAS TB-IGRA has higher sensitivity while maintaining specificity and leads to fewer indeterminate results than QFT-Plus (18).

QFT-Plus can be a good tool for detecting LTBI that is not detected by the Mantoux test or Diaskintest tests, as well as for refuting false-positive skin test results, particularly those occurring in children after BCG vaccination. However, the use of QFT-Plus in screening for MBT infection must be accompanied by other testing methods (19).

Early detection of LTBI in children is of great importance. According to Iranian authors, LTBI screening in children in countries with a high burden of tuberculosis is in some cases limited by a lack of resources. They conducted a study of 230 children with family contact with tuberculosis, who were diagnosed with LTBI using a skin tuberculin test and QuantiFERON^®^-TB Gold Plus at the time of contact detection and then at 3, 12, and 18 months. The study found LTBI in 45.2% of children with documented family contact. Such a high level of LTBI is alarming and indicates the need for more in-depth measures to diagnose LTBI (20).

Immunological diagnostic methods are particularly important in individuals with immunosuppression (21). Numerous studies in recent years have focused on comparing the diagnostic significance of the immunological tests used (22). In some studies, the authors point to the need to use several tests simultaneously, due to the uncertain results of IGRA tests. Italian authors published a study evaluating the effectiveness of T-SPOT.TB in patients with indeterminate QuantiFERON-TB Gold Plus results. Of the 137 patients with an indeterminate QuantiFERON-TB Gold Plus result, T-SPOT.TB provided a definitive result in 120 patients (87.6%), of whom 80 were negative and 40 were positive. The authors suggest performing T-SPOT.TB within 30 days after receiving an indeterminate QuantiFERON-TB Gold Plus result as a possible new algorithm for diagnosing LTBI (23). Skin tests using only two specific antigens, ESAT-6 and CFP-10, are also becoming more widespread, for example, Diaskintest^®^ (Russia), C-Tb (Denmark) and CTb C-TST (formerly known as the ESAT6-CFP10 test (China) (9, 24, 25). Several studies have compared QFT-Plus with QFT-GIT, T-SPOT.TB, and tuberculin tests in different groups (26, 27). In 2022 the WHO issued an information bulletin entitled “Rapid communication: TB antigen-based skin tests for the diagnosis of TB infection,” in which it officially referred to studies conducted on new skin tests, such as C-Tb (Serum Institute of India, India), C-TST (formerly known as the ESAT6-CFP10 test, Anhui Zhifei Longcom, China), Diaskintest^®^ (Generion, Russian Federation), their diagnostic significance and safety profiles. Undoubtedly, the WHO’s approval of these tests, including the Russian Diaskintest^®^, has significantly contributed to their use worldwide (16, 24, 28). The diagnostic parameters of Diaskintest have been evaluated in various studies and presented in a meta-analysis (29, 30). Belarusian authors conducted a comparative analysis of three tests: QFT-Plus, Mantoux test, and Diaskintest using the example of LTBI diagnosis in 41 patients. They obtained the following results: QFT-Plus and Mantoux test had a satisfactory degree of agreement (kappa 40.21–0.40), however, in individuals under 18 years of age, the agreement between these tests was insignificant (kappa 0.10–0.20). QFT-Plus and Diaskintest had a moderate degree of agreement (kappa 0.41–0.60). Among the non-concordant results of QFT-Plus and the Mantoux test (*N* = 16), discrepancies were more often observed in the direction of a positive Mantoux test and a negative QFT-Plus result. Among the discrepancies between QFT-Plus and Diaskintest, no clear trend in either direction was identified (19). However, all of the tests presented have a number of limitations, including the inability to differentiate between active tuberculosis and LTBI, false positive results in individuals vaccinated with BCG (only for the tuberculin test), false negative results in children, the elderly, and immunocompromised patients, and the inability to predict the progression of LTBI to active tuberculosis (16, 29, 31).

IP-10 (CXCL10) and HBHA-IGRA are being studied for research purposes and may be useful for assessing the risk of latent infection progression and monitoring therapy (32, 33). However, these methods have not yet been widely implemented in clinical practice.

Antigen-specific skin tests demonstrate high specificity thanks to the use of ESAT-6 and CFP-10 antigens, excluding cross-reactions with BCG and most non-tuberculous mycobacteria (24). Despite this, their prognostic value is comparable to that of IGRA: a positive result only indicates the presence of infection, but does not allow for reliable prediction of progression. Russian data show a possible correlation between the severity of infiltrate in the Diaskintest and the risk of infection activation, but this indicator is not standardized and is not included in international recommendations. In 2024, a new IGRA-TB (Russia) was registered in the Russian Federation. The test uses peptides to stimulate CD4 + lymphocytes and CD8 + in a single tube for immunoenzymatic determination of interferon-gamma in blood plasma isolated from heparinized whole human blood to determine the specific T-cell response. The study proved that the test has high diagnostic parameters comparable to QuantiFERON-TB Gold Plus. Decentralized platforms (e.g., QIAreach™ QuantiFERON) reproduce the results of laboratory IGRA, retaining their diagnostic and prognostic limitations. Their advantage lies more in expanding access to screening than in improving predictive accuracy.

### 4.1 Comparative analysis and limitations of immunological methods in the diagnosis of tuberculosis infection

Interferon-gamma release assays (IGRAs) demonstrate higher specificity than the tuberculin skin test (TST) in Bacillus Calmette–Guérin (BCG)-vaccinated populations and require only a single patient visit. However, neither IGRAs nor TSTs can reliably distinguish latent *Mycobacterium tuberculosis* infection from active disease, and both have limited predictive value for progression to active tuberculosis. Emerging immunological biomarkers, including IP-10 and transcriptional signatures, show promise for improving diagnostic accuracy and disease prediction, yet require further standardization and validation before broad implementability to Distinguish Latent and Active Infection. Both TST and IGRA detect host immune sensitization to M. tuberculosis antigens, but do not indicate the presence of active bacterial replication. A positive result confirms exposure rather than active disease. TST nor IGRA reliably predict progression from latent infection to active TB, leading to limited utility in targeted preventive therapy. TST reactivity is affected by prior BCG vaccination and environmental mycobacterial exposure. In contrast, IGRAs use M. tuberculosis-specific antigens (ESAT-6, CFP-10), providing higher specificity in BCG-vaccinated populations. Both assays show reduced sensitivity in individuals with low CD4 counts or receiving immunosuppressive therapy. The proportion of indeterminate IGRA results increases in advanced HIV infection. In young children (<5 years), both tests show variable sensitivity and require clinical, radiological, and microbiological correlation. TST remains inexpensive but requires two visits and subjective interpretation. IGRAs, while more specific, are cost-intensive, time-sensitive (blood processing within 6–16 h), and depend on laboratory infrastructure, limiting scalability in low-resource settings. IGRA results close to the threshold may fluctuate between positive and negative upon repeat testing; careful interpretation is required, especially for serial screening programs. IP-10 assays show pooled sensitivity of ∼72–86% and specificity of 83–88% across meta-analyses, suggesting utility in settings with high immune activation (e.g., HIV infection), though standardization is lacking. Transcriptional signatures (3–16 gene sets) have demonstrated predictive potential for incipient TB, with AUCs 0.7–0.9, but still require validation and assessment of cost-effectiveness for programmatic use station (Programmatic Advertising Platforms).

### 4.2 Predictive value of tests

According to a meta-analysis by the WHO and CDC (10), positive results for both TST and IGRA are associated with a higher risk of progression of tuberculosis infection to active tuberculosis. The relative risk is approximately 2–4 compared to individuals with negative tests. However, the absolute risk is low: among IGRA-positive individuals without treatment, active tuberculosis develops in approximately 2–3% within 2 years of follow-up. Thus, both methods have limited prognostic value and do not allow for the reliable identification of the group of patients at highest risk. IP-10 (CXCL10) has shown higher sensitivity in children, HIV-infected individuals, and immunosuppressed patients. It is currently being considered as an alternative or supplement to IGRA, as well as a potential marker of progression risk (32). IL-2 and the IFN-γ/IL-2 ratio are more often associated with latent infection, while IFN-γ is associated with active inflammation. However, the IFN-γ/IL-2 ratio may be an indicator of the stage of infection (LTBI vs. active TB) (34). Combinations of TNF-α, GM-CSF, and IL-17 markers are being studied as part of multicytokine panels. Their combination with IFN-γ and IL-2 increases the sensitivity and specificity of LTBI diagnosis (35). The antigen-specific marker HBHA (heparin-binding hemagglutinin adhesin)-IGRA is considered a candidate for differentiating LTBI and active tuberculosis (33). ESAT-6/CFP-10 in combination with other antigens in the form of expanded antigen panels (e.g., including TB7.7, PPE peptides) may improve LTBI detection in different populations (36) (Table 1).

**TABLE 1:** Comparative characteristics of modern immunological tests for the diagnosis of tuberculosis infection.

Test	Principle	Time to result	Sensitivity/specificity (according to studies)
VIDAS TB-IGRA	IFN-γ (ELFA)	∼ 17 h	Sensitivity 94–96%, specificity ∼97% (14)
LIAISON QFT-Plus	IFN-γ (CLIA)	∼ 16–20 h	Comparable to QFT-Plus
QIAreach QFT	IFN-γ (immunochromatography)	20–30 min	Sensitivity ∼99%, specificity ∼94%
C-TST/C-Tb/Diaskintest	Skin test (ESAT-6/CFP-10)	48–72 h	Sensitivity 85–90%, specificity 95–98% (24)
IP-10/ HBHA-IGRA (under investigation)	Cytokine response (IP-10, HBHA)	24–48 h	Promising high sensitivity in immunocompromised individuals (32)

In the UK, as part of the UK PREDICT TB study published in 2018, 9,610 people were screened to compare the predictive value of tuberculin skin tests with IGRA tests in individuals with LTBI for the development of active tuberculosis. All were screened using QuantiFERON-TB Gold-In Tube, T-SPOT.TB, and tuberculin skin test. The annual incidence among participants with positive test results was highest for T-SPOT.TB, followed by TST-15 and then QuantiFERON-TB Gold In-Tube in descending order, reflecting the predictive value of these tests (38). The same study evaluated the predictive value of tests and combinations of tests in identifying individuals who would subsequently progress to active tuberculosis (Table 2). Various combinations of immunological tests were used for this purpose, such as the tuberculin skin test and T-SPOT.TB, the tuberculin skin test and QuantiFERON^®^ TB Gold In-Tube, etc. This study found minor differences between tests or combinations of tests in identifying individuals who would subsequently develop active tuberculosis. However, a two-step approach combining a tuberculin skin test with BCG stratification and IGRA proved to be the most cost-effective testing option.

**TABLE 2:** Prognostic significance of immunological tests for the diagnosis of tuberculosis infection.

Test	Relative risk of progression (compared to negative test)	Absolute risk of active tuberculosis over 2 years with a positive result	Features
TST	∼2–3 (10)	2–3%	Affected by BCG, limited prognostic value
IGRA (QFT, T-SPOT)	∼2–4 (10)	2–3%	High specificity, but limited prognostic accuracy
TBST (C-TST, C-Tb, **Диаскинтест**^®^)	Data are limited; comparable to IGRA (24)	2–3%	Excludes the influence of BCG; the relationship between infiltrate size and risk of activation requires standardization.
Point-of-care IGRA (QIAreach QFT)	Similar to IGRA	∼2–3%	Prognostic value no higher than laboratory IGRA
IP-10 (CXCL10)	Higher levels are associated with a higher risk (32)	No precise data available	At the research stage, promising for prognosis
HBHA-IGRA	HBHA-specific response is associated with low risk of progression (37)	No precise data available	Possible marker of stable latent infection, currently under investigation

### 4.3 Cell markers and phenotypes of T cells

**FIGURE 1:**
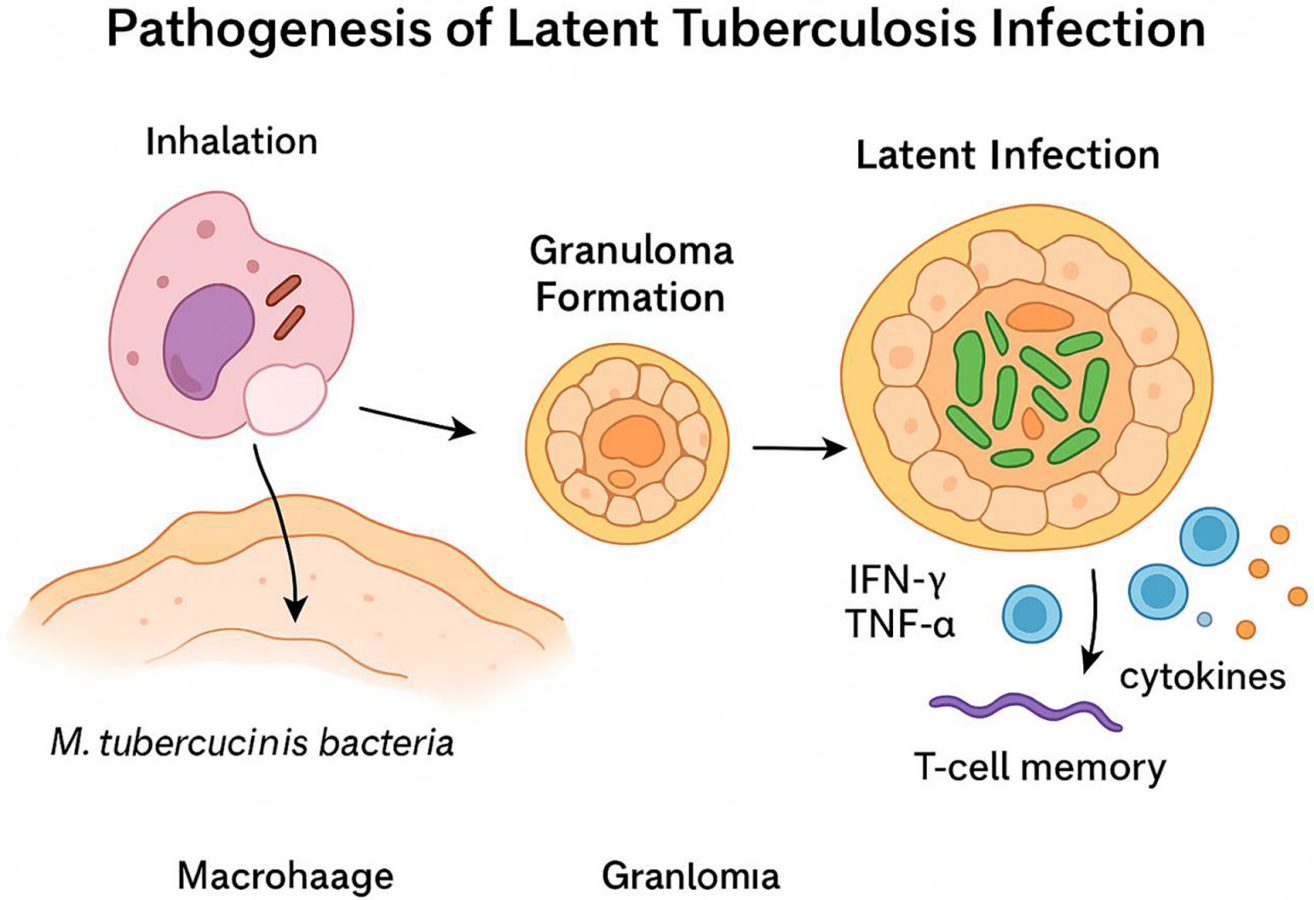
Pathogenesis of latent tuberculosis infection.

The diagnosis of latent tuberculosis infection (LTBI) is traditionally based on immunological tests such as the tuberculin skin test (TST) and interferon-γ release assays (IGRA). However, these methods only reflect the fact that the immune system is sensitized to *Mycobacterium tuberculosis* (Mtb), without allowing a reliable distinction between latent status, active disease, or assessment of the risk of reactivation (Figure 1). In this regard, researchers are focusing on a more subtle level—the characterization of phenotypes and functional activity of T lymphocytes involved in the immune response to Mtb (27). In this regard, new biological markers are being actively researched (26, 27).

Host immune biomarkers that are specifically and differentially expressed during contact or infection have become an attractive prospect for the detection and diagnosis of tuberculosis. Research: expression of CD27, CD38, HLA-DR, and Ki-67 on Mtb-specific CD4 + T cells allows differentiation between LTBI, active TB, and completed treatment (39). HLA-DR and CD38 on antigen-specific T cells have been identified as stable markers for distinguishing LTBI from active tuberculosis (39, 40). The combination of immune interactions ultimately determines the outcome of infection, allowing or preventing primary infection, triggering the immune response, and influencing the outcome of each individual granuloma (41). Multiparametric approaches are a promising avenue. IP-10 (CXCL10) levels and response to HBHA antigen have shown a closer association with the risk of progression. The HBHA-specific T-cell response is considered a marker of stable latent infection (low risk of progression), while elevated IP-10 levels may be an indicator of a higher likelihood of transition to active disease (32, 37). However, the data remain experimental, and the tests have not yet been implemented in clinical practice. Individuals with LTBI are more likely to have multifunctional CD4^+^ T cells that simultaneously produce IFN-γ, TNF-α, and IL-2. This profile is considered a sign of balanced infection control. In active tuberculosis, there is a predominance of “differentiated” T cells with limited functionality (e.g., IFN-γ^+^/TNF-α^+^, but without IL-2). The CD27^+^/CD27^–^ ratio among Mtb-specific CD4^+^ T cells is also proposed as a potential marker: loss of CD27 is associated with active disease. However, a positive IGRA/TST alone does not distinguish between recent infection, distant latency, or risk of progression (42, 43). Multifunctional T cells (simultaneous production of IFN-γ, IL-2, TNF-α) and distribution across memory subpopulations (T_CM, T_EM, T_SCM, T_RM) are being studied as markers of response quality; local pulmonary T_RM are particularly relevant for control at the site of infection. Immunological characteristics: expression of activation/differentiation markers on Mtb-specific CD4 + cells (CD27↓, CD38 + , HLA-DR + , Ki-67 + ) is consistently associated with active TB and helps to distinguish ATB from LTBI; several studies and reviews show good reproducibility of these markers in diagnostic panels (37). PD-1, TIGIT and other markers of T cell exhaustion are being studied as signs of loss of control over Mtb. Increased PD-1 expression on Mtb-specific CD4^+^ T cells is more commonly seen in active tuberculosis. Th1-type CD4 T cells are thought to play a crucial role in the protective immune response to M. tuberculosis. They are likely supported by IL-17-producing CD4 T cells, non-traditional T cells, and CD8 T cells that secrete cytokines and exhibit cytolytic activity (41). Promising biomarkers are a combination of phenotypes (CD27, CCR7, CD45RA) with cytokine production profiles (IFN-γ, TNF-α, IL-2), which allow for more accurate differentiation between LTBI and active disease. The use of multiparametric flow cytometry and single-cell RNA-seq technologies opens up opportunities for creating prognostic “immune signatures” of reactivation risk (44, 45). Polyfunctional T cells that secrete IFN-γ, IL-2, and TNF-α simultaneously are associated with infection control. CD27 low CD4 + T cells are more commonly detected in active TB and may serve as a marker for differentiation from LTBI (46). T-SCM (stem-cell-like memory T cells) and T_RM (tissue-resident memory T cells) are considered to be the immunological basis of long-term latent infection (47). It is known that increased expression of IFN-γ and TNF-α is a cytokine correlate of tuberculosis. It has been shown that its increase in contacts can serve as a predictor of tuberculosis development (48). Gene expression panels (transcriptomic signatures, e.g., RISK11) can predict the progression of LTBI to active tuberculosis within 6–12 months (49). Metabolic markers (e.g., lipid and metabolite profiles associated with inflammation, as well as vitamin D) are in the early stages of study but show promise in differentiating stages of infection. As is well known, the mechanism of action of IGRA tests, Diaskintest^®^, and the C-TB skin test is based on the use of the proteins ESAT-6 and CFP-10. However, there are other proteins that can be used to develop tests that can more accurately distinguish LTBI from post-vaccination allergy after BCG immunization, as well as LTBI from active tuberculosis. M. tuberculosis antigens associated with LTBI-RD, such as Rv1736c, Rv1737c, Rv2031c, Rv2626c, Rv2653c-Rv2660c, etc., are a source of proteins that can be used to detect LTBI in isolation. With regard to the host organism, cytokines IL-2, IL-10, IP-10 and VEGF, MCP-2, fractalkine, granzyme B, etc. are of interest, as they can presumably be used in the development of new tests for the differential diagnosis of LTBI and active tuberculosi. (42). Garlant et al. (43). conducted a study of 9 expressed proteins CALCOCO2, CD274, CD52, GBP1, IFIT3, IFITM3, SAMD9L, SNX10, and TMEM49 using enzyme-linked immunosorbent assay (ELISA). Based on these data, it is possible to diagnose various stages of the infectious process in tuberculosis. The most effective single biomarkers for pulmonary tuberculosis, compared to control groups, were CALCOCO2, SAMD9L, GBP1, IFITM3, IFIT3, and SNX10. The creation of tests based on protein data may be of significant diagnostic value and simplify the identification of patients with LTBI (43). Drain et al. provide data on the presence of a correlation observed in the initial and subclinical course of tuberculosis (50). Scriba et al. (51) point to an increase in IFN levels during the progression of tuberculosis infection, which is understood as the transition from LTBI and subclinical forms of tuberculosis to active forms. They also observed an increase in IgG and IgA levels in so-called “progressors,” a decrease in B- and T-cell signaling, and activation of myeloid cell functions, including phagocytosis, several months before the onset of clinical signs of tuberculosis (51, 52). Understanding the underlying mechanisms of interaction between the host immune system and *M. tuberculosis* helps to identify targets for specific and non-specific tuberculosis prevention (Table 3).

**TABLE 3:** New directions and key markers.

Key markers	Applications
Plasma biomarkers	GM-CSF, CXCL10, IL-1Ra—high prognostic AUC ≥ 90%
Lung cells (T_RM, KLRG1 + )	Lung cells (T_RM, KLRG1 + ) In progressors—local CD4 + T_RM and KLRG1 +
miRNA panel (7 miRNAs)	miRNA panel (7 miRNAs) Prediction of LTBI → ATB transition (in QuantiFERON supernatants)
RNA signature (16 genes)	Progression prediction with ∼71% sensitivity 6 months prior to diagnosis
Changes after prevention	Decreased IFN-gene signatures in the risk group
Multiomics	Transcriptomics + metabolomics = improved prognostic accuracy

Identifying factors affecting the body’s reactivity that influence false-negative results remains an important task in improving the interpretation of IGRA test results. A study by Santos et al. reports on IGRA tests and tuberculin skin tests performed on 727 patients with active tuberculosis. Sensitivity was 82.4, 84.6 and 78.4% for IGRA, TST-5 mm and TST-10 mm, respectively. These results indicate that 17.6, 15.4 and 21.6% of patients diagnosed with tuberculosis had false-negative results for IGRA, TST-5 mm and TST-10 mm, respectively. According to the study, the highest proportion of indeterminate IGRA test results increased in patients over 80 years of age and the lowest in patients under 20 years of age. Thus, it was shown that age can be a predictor of indeterminate or false-negative results (53).

In China, a retrospective study was conducted on children and adolescents under 18 years of age from a risk group for concomitant disease for the presence of LTBI using X.DOT-TB (44.9% of those examined) and QuantiFERON-TB Gold In-Tube (15% of those examined). The researchers encountered certain difficulties in interpreting the results, namely, they encountered a large number of indeterminate results in children with respiratory and rheumatic diseases. As the authors write, immunosuppressants used in rheumatic diseases can cause lymphopenia or impair the function of T cells or antigen-presenting cells, which can significantly affect the uncertainty of the results. Thus, the presence of comorbidities demonstrated a statistically significant association with IGRA indeterminate results (54). Although positive IGRA test results have high predictive value for the development of tuberculosis, false-negative results remain a problem. Among 274 patients with established tuberculosis in a study by Li et al., 80.7% were IGRA-positive and 19.3% were IGRA-negative. The researchers identified a correlation between older age and negative IGRA test results. False-negative IGRA results remain an unresolved diagnostic dilemma that requires a high index of clinical suspicion. Another interesting observation was elevated IL-4 levels and decreased IFN-γ, IL-2, IL-6, IL-1β, and IL-12 levels in IGRA-negative tuberculosis compared to the IGRA-positive tuberculosis group (55). Thus, external validation of transcriptomic and cellular panels in different populations, within a single population, and taking into account the spread of tuberculosis infection is currently needed. Immunosuppression and HIV infection must be taken into account. An important factor is the age of patients, which can affect the evaluation of test results (e.g., children and the elderly). It is practically relevant to combine multi-omic approaches (transcriptome, epigenome, metabolome, cell phenotype) to develop clinically applicable tests for the risk of progression. Such information will be extremely relevant for consideration and application in groups at risk of developing LTBI and further active tuberculosis.

## 5 Risk groups

The results of LTIs detection in risk groups demonstrate that its prevalence is significantly higher among contacts, HIV-infected individuals, children, and migrants from endemic regions. The greatest prognostic significance of LTBI detection is observed in HIV-positive patients, young children, and individuals receiving immunosuppressive therapy, confirming the need for targeted screening and preventive treatment in these groups. WHO recommendations identify population categories that should be considered at risk for developing tuberculosis (8). Briefly, the main groups for which the World Health Organization recommends regular monitoring for LTBI are:

- HIV-infected individuals, including children;
- patients starting therapy with TNF inhibitors;
- patients on dialysis;
- patients preparing for organ transplantation or blood transfusion;
- patients with silicosis;
- prisoners, healthcare workers, immigrants from countries with a high burden of tuberculosis, homeless people and people who use illicit drugs (for countries with low tuberculosis incidence);
- children under 5 years of age who have been in contact with patients with pulmonary tuberculosis at home;
- adults, adolescents and children who have been in contact with patients with respiratory tuberculosis at home (for countries with low tuberculosis incidence);

Table 4 presents data from the literature on the prevalence of LTBI in risk groups.

**TABLE 4:** Prevalence of LTBI in risk groups.

Gisk groups	Prevalence of LTBI (IGRA/TBST positive)	Progression risk characteristics
Close contacts of patients with TB	30–50%	Highest risk of progression within the first 2 years
HIV-infected individuals	10–40%	High likelihood of progression to active TB with low CD4 counts
Patients receiving TNF-α inhibitors	10–30%	Very high risk of reactivation; mandatory screening required
Healthcare workers	5–40% (depending on regional TB burden)	Occupational risk; regular screening recommended
Children (contacts)	20–30%	Highest risk of progression in children < 5 years
Migrants from endemic countries	20–35%	Screening recommended upon entry to low-incidence countries

### 5.1 Close contacts of patients with TB

The duration of contact with the so-called index case influences the results of immunological tests for detecting LTIs. In Catalonia, Spain, a study of immigrants shows that the risk of LTBI increases in individuals who have had prolonged contact. LTBI was confirmed by a positive tuberculin skin test and IGRA test, followed by further examination to exclude individuals with active tuberculosis from the study. The study showed that the risk of LTBI associated with exposure ≥ 6 h/day and < 6 h/day but ≥ 6 h/week was 2.0 and 1.6 times higher than with exposure < 6 h/week (56). The correlation between contact duration and the risk of developing LTBI is also indicated by the authors Choi Y, Park SJ, An HS et al. from South Korea in an epidemiological investigation of a tuberculosis outbreak in a school. Contacts at the school were screened using the IGRA test, chest X-rays and/or chest CT scans. Genotyping of Mycobacterium tuberculosis isolates was also performed using whole genome sequencing (WGS). In the course of identifying individuals with LTBI and tuberculosis patients, the authors concluded that prolonged exposure exceeding 10 h per week was associated with a significant increase in the risk of tuberculosis infection (57). Reichler et al. (58) assessed the influence of various factors on the likelihood of LTBI, as determined by a tuberculin skin test, in contacts. Among the factors they analyzed, they highlighted contact duration of more than 5 years, Asian or Latin American race/ethnicity and foreign birthplace of the contact, MBT ( + ) in the patient with whom contact occurred, bilateral process and presence of CV( + ) cavity, household contact, and contact with more than one tuberculosis patient. They also analyzed the duration of contact, measured in hours, and found that the prevalence of LTBI was higher among those who had been in contact for more than 250 h, with the probability of LTBI increasing by 8.2% for every additional 250 h spent in close contact. However, among individuals with contact duration of less than 250 h (the threshold number), the prevalence of LTBI was lower and no longer correlated (for example, the prevalence of LTBI with contact duration of 50 h or 150 h was approximately the same). This is obviously an important observation, since knowing the duration of contact allows this information to be used to predict the likely level of LTIs when testing contacts (58). In Portugal, there was a gradual shift in the national strategy for LTBI diagnosis from tuberculin skin testing to IGRA testing in 2016. A comparison was made between 499 contacts who underwent both the tuberculin skin test and the IGRA test (interim transition phase) and 547 contacts who underwent only the IGRA test (final transition phase). The results showed that performing only IGRA tests was more cost-effective than the two-stage testing strategy (59). The example of Moscow (Russia) showed that the use of only one immunological test does not provide a clear answer as to the presence or absence of LTBI (60). According to Indian recommendations, treatment of children under 5 years of age who have been in contact with tuberculosis patients in the family is recommended after ruling out active tuberculosis, regardless of the results of tuberculosis testing. However, the authors emphasize that the true level of LTBI in the country is unknown. In view of this, a study of 369 children under the age of 5 was conducted in Mumbai to detect LTBI using a tuberculin skin test and an IGRA test. The overall prevalence of LTBI among children under the age of five was 12.4% according to IGRA and 21.4% according to the tuberculin skin test. Since LTBI was not detected in all of the children studied, the authors believe that it is necessary to adhere to an approach whereby the child is first tested and then, based on the test results, preventive chemotherapy is prescribed (61). In the Russian Federation, this issue is regulated by clinical guidelines, which stipulate the need for examination and observation of contacts, especially children, over a long period of time with a comprehensive examination (62).

### 5.2 People living with HIV infection

Patients with HIV infection are at the highest risk of progression from latent tuberculosis infection (LTBI) to active disease, with a lifetime risk of approximately 30% compared to about 10% in the general population. The diagnosis of LTBI in individuals with HIV infection has received considerable attention, as the risk of progression from LTBI to active TB in this group is increased by 50–200-fold. This risk remains high even among patients receiving antiretroviral therapy (63). According to various studies, the prevalence of LTBI among HIV-infected individuals varies substantially depending on the immunological test applied. HIV-positive individuals demonstrate significantly lower responsiveness to both IGRA and the tuberculin skin test (TST). A high level of discordance has been reported between different generations of IGRA (QuantiFERON-TB Gold and QuantiFERON-TB Gold In-Tube), ELISPOT, and skin tests, which is attributed to immune energy in HIV-infected patients and to the diagnostic capacities of the assays (64–66). A meta-analysis of 37 studies involving 5736 HIV-infected individuals showed that immunosuppression exerted less impact on ELISPOT compared to QFT-GIT and the TST (67). It is important to consider the degree of immunosuppression when diagnosing LTBI in immunocompromised patients. In individuals with CD4 + T-lymphocyte counts above 350 cells/μL, any modern immunodiagnostic method can be applied, whereas the diagnostic value of skin tests decreases with lower CD4 levels (66, 68). In the natural course of infection caused by the *Mycobacterium tuberculosis* complex, CD4 + T-lymphocytes play a critical role in immune control due to their ability to secrete IFN-γ. Evidence has also been obtained supporting the role of CD8 + T-lymphocytes in host defense against *M. tuberculosis* complex through IFN-γ production and other mechanisms that activate macrophages, suppress mycobacterial growth, eliminate infected cells, or mediate direct lysis of intracellular mycobacteria. Specific CD8 + T-lymphocytes have been detected in individuals with LTBI or active TB, with higher frequencies of IFN-γ–producing CD8 + T cells observed in those with active disease. Moreover, CD8 + T-lymphocytes specific to ESAT-6 and CFP-10 are more frequently found in patients with active TB than in those with LTBI, likely reflecting recent exposure to *M. tuberculosis*. Furthermore, IFN-γ–producing CD8 + T-lymphocytes have been reported in patients with TB and concurrent HIV infection, as well as in young children with TB (69, 70). According to a multicenter Italian study (TUBHIVIT), the prevalence of LTBI among people living with HIV ranged between 2.8 and 11.2% (69–71). The influence of HIV-induced immunosuppression on the performance of immunological tests is of particular interest. Petruccioli et al. (72) reported that HIV infection did not affect QFT-Plus results in active TB and that CD4 counts did not influence the distribution of IFN-γ responses in patients with HIV-TB and HIV-LTBI. However, the authors observed that HIV infection impacted CD4 + T-cell responses to QFT-Plus, while CD8 + T-cell responses remained similar between HIV-infected and non-infected individuals. These findings are especially important, as they indicate that the TB2 stimulation component of the assay remains unaffected in people living with HIV. It is likely that the CD8-specific response compensates for the impaired CD4 response associated with HIV infection, thereby ensuring comparable sensitivity of QFT-Plus in HIV-infected and HIV-uninfected populations (72).

### 5.3 Healthcare workers

It should be emphasized that healthcare workers (HCWs) also belong to a recognized risk group for the development of tuberculosis. The ratio of TB risk among HCWs compared with that of the general adult population is one of the indicators recommended by the WHO for assessing the impact of infection prevention and control measures in healthcare facilities. When preventive measures are effective, the relative risk of TB among HCWs compared to the general population should approach unity. In 2023, a total of 17,449 cases of tuberculosis among HCWs were reported from 73 countries (3). The mean 5-year prevalence of LTBI among HCWs was 3.3%, underscoring the role of individuals with LTBI as a potential reservoir for progression to active TB. This finding also highlights the rationale for implementing preventive therapy among high-risk groups, including HCWs (73, 74). In certain countries, however, LTBI prevalence among HCWs has been reported as high as 47% (75). The correlation between TST and IGRA was evaluated among 266 medical students in Bandung, Indonesia, where 31.9% had a positive TST and 16.2% were IGRA-positive. Agreement, calculated using Cohen’s kappa coefficient, was 74.7%. Interestingly, students with household contact with TB cases were more likely to have negative test results, demonstrating a paradoxical inverse correlation between exposure and test positivity (76). Several countries have developed specific strategies for TB prevention among HCWs. In Brazil, a study was conducted among hospital employees over the period 2005–2018, aiming to compare TB incidence before and after the implementation of a TB prevention strategy in 2012. Between 2005 and 2011, the incidence among HCWs was 100 per 100,000, which decreased to 26.2 per 100,000 following the introduction of preventive measures in 2012–2018. These findings illustrate the potential effectiveness of innovative approaches to LTBI diagnosis and the identification of high-risk subgroups among HCWs (77). In Peru, a study was conducted in 2022–2023 among healthcare staff, all of whom underwent IGRA testing for LTBI. LTBI was defined as a positive IGRA result in the absence of clinical or radiological abnormalities. After screening 308 staff members and performing multivariate analysis, LTBI prevalence was estimated at 17.86%. Male staff members and those with longer professional experience (>10 years) were at particularly increased risk (78). Of particular interest is the Italian CROSSWORD study, which aims to evaluate LTBI prevalence among HCWs and medical trainees in hospital settings, with the goal of identifying risk factors such as sex, age, BCG vaccination history, profession (physician, nurse, student), duration of employment, and interferon response levels. The ultimate objective of the study is the development of a web-based platform to predict LTBI risk. According to the authors, results are expected to be available in 2026 (79). Istomina and colleagues conducted a study stratifying participants into three groups: HCWs in specialized TB facilities, HCWs in general healthcare settings, and a comparison group of healthy individuals without TB contact. LTBI was diagnosed using the recombinant tuberculosis allergen skin test (Diaskintest)^®^. The prevalence of LTBI among HCWs in general healthcare facilities was found to be similar to that in healthy controls. Notably, infection rates were higher in departments of TB facilities treating pulmonary TB compared with departments managing extrapulmonary TB or administrative staff. These findings provide a basis for stratifying departments within TB facilities into categories of low, medium, and high LTBI risk (80).

Another study assessed LTBI in HCWs using multiple diagnostic methods, including the Mantoux test (2 TU), Diaskintest^®^, QuantiFERON-TB, and T-SPOT.TB. Results indicated comparable diagnostic utility of all assays except for the Mantoux test, which demonstrated reduced diagnostic accuracy (81). In the Tyumen region of Russia, LTBI prevalence was assessed among staff of the forensic medical examination bureau using Diaskintest^®^. LTBI was 3.5-fold higher in Group 1 (exposed staff) compared with Group 2 (controls). LTBI was diagnosed in one-third of employees, and more than half of them demonstrated pulmonary calcifications on chest CT (82). In Tajikistan, a study was conducted among 364 staff of the National Center for Tuberculosis, Pulmonology, and Thoracic Surgery in Dushanbe. LTBI was diagnosed using the Mantoux test (2 TU PPD-L), Diaskintest^®^, and QuantiFERON testing. Following additional evaluation, active TB was confirmed in two employees, while two others had residual radiological changes. Ultimately, 135 employees (37.1%) were diagnosed with LTBI due to the absence of clinical manifestations. This high prevalence underscores the need for specific TB prevention programs for HCWs. Interestingly, vitamin D deficiency was detected in more than 80% of participants (83). In Bangladesh, a comparative study evaluated QuantiFERON-TB Gold Plus against the more affordable E TB Feron ELISA test, widely used in the region. Participants were stratified into four groups: healthy controls, HCWs/caregivers of TB patients, microbiologically confirmed TB patients, and individuals with a history of TB. Concordance was assessed using positive percent agreement (PPA), negative percent agreement (NPA), and Cohen’s kappa coefficient. Indeterminate results were excluded from the analysis, and overall agreement reached 85.9% (84). Healthcare workers represent a population at elevated risk of both latent and active tuberculosis due to occupational exposure. The heterogeneity of LTBI prevalence among HCWs across different countries highlights the influence of local epidemiology, diagnostic approaches, and the effectiveness of infection control measures. Evidence from multicenter studies demonstrates that preventive interventions can substantially reduce TB incidence among HCWs, while discrepancies in test concordance underscore the need for careful selection of diagnostic tools. The identification of subgroups with particularly high occupational risk, including staff in pulmonary TB units and individuals with long-term employment, further supports the rationale for targeted LTBI screening and preventive therapy. Collectively, these data emphasize the importance of implementing comprehensive TB prevention and control strategies specifically tailored to healthcare settings.

### 5.4 Patients on hemodialysis

A distinct subgroup among high-risk populations comprises individuals undergoing hemodialysis. Patients with end-stage renal disease (ESRD) receiving hemodialysis are at markedly increased risk of developing active tuberculosis. The prevalence of LTBI in this group reaches 25–35% when assessed by IGRA, whereas TST often underestimates infection due to energy and prior BCG vaccination (85). Patients with ESRD experience profound immune dysfunction involving both innate and adaptive responses. Impaired T-lymphocyte function constitutes the main deficit: uremia reduces proliferative activity and disrupts the synthesis of key cytokines, particularly interferon-gamma (IFN-γ), which is a central marker in TB immunodiagnostics (IGRAs). In addition, monocyte/macrophage dysfunction decreases antigen-presenting capacity and diminishes phagocytic efficiency. Cell-mediated immunodeficiency in ESRD increases the risk of progression to active TB by 8–25 times compared with the general population. LTBI screening is recommended at the initiation of dialysis and annually thereafter, with IGRA as the preferred diagnostic tool. In cases of confirmed LTBI, preventive chemotherapy is advisable, often administered under directly observed therapy during dialysis sessions to enhance treatment completion rates (86). In Japan, a study assessed LTBI prevalence among patients with chronic kidney disease (CKD) on dialysis. A total of 118 patients were tested using IGRA. Some studies indicate that T-SPOT.TB demonstrates slightly higher sensitivity and fewer indeterminate results in immunocompromised patients compared with QuantiFERON-TB, although the data remain inconsistent. None of the patients had active TB at the time of evaluation; LTBI was not detected in 96 patients, while in 8 cases results were inconclusive. LTBI was confirmed in 14 patients. Moreover, a higher degree of nephrosclerosis was associated with an increased likelihood of LTBI (87, 88). Patients with end-stage renal disease undergoing hemodialysis represent a population at substantially elevated risk of LTBI and its progression to active tuberculosis, owing to profound defects in both innate and adaptive immunity. IGRA testing is preferred for screening in this group, as TST frequently underestimates infection due to immune energy and prior BCG vaccination. Regular LTBI screening at dialysis initiation and during follow-up, combined with timely preventive therapy, is essential to reduce the risk of active TB. Emerging evidence also suggests an association between the degree of renal pathology, such as nephrosclerosis, and LTBI prevalence, highlighting the need for further research and tailored preventive strategies in this vulnerable population.

### 5.5 Diabetes mellitus

Several studies have demonstrated a positive association between LTBI and diabetes mellitus (DM), indicating that individuals with DM are more susceptible to LTBI and, consequently, to progression toward active tuberculosis. A meta-analysis of 22 studies (∼68,000 participants) showed that people with diabetes have an increased risk of LTBI, with an adjusted OR of approximately 1.21 (95% CI 1.14–1.29), while three cohort studies reported a pooled aRR of ∼1.26 (95% CI 0.71–2.23) (89). Latent tuberculosis infection (LTBI) screening using immunological assays shows substantial variability across patient populations with chronic immune dysregulation. For example, in a study conducted in Sana’a, Yemen (2023), among 150 patients with type 2 diabetes mellitus (T2DM), LTBI prevalence reached 29.3%—25.3% by IGRA and 21.3% by TST—with a high concordance between the two tests (κ = 0.67; 88% agreement). Similarly, in a large cohort of 5224 patients with rheumatic diseases evaluated before TNF inhibitor therapy, positive rates for TST, QuantiFERON-TB Gold In-Tube, and T-SPOT.TB were 29, 17, and 18%, respectively, with concordance levels between 73 and 75%. Taken together, these data highlight that both metabolic and autoimmune conditions can affect the performance and concordance of immunodiagnostic assays, likely due to underlying immune modulation. However, differences in study design, geographic setting, and immune status of the cohorts limit the comparability and generalizability of findings. Larger, standardized studies should to clarify the diagnostic reliability of IGRAs and TST across diverse immunocompromised populations (90–92).IGRA tests maintain diagnostic sensitivity in diabetic patients. For instance, in studies involving active TB patients, QuantiFERON-TB Gold demonstrated 81% sensitivity in diabetic individuals versus 63% in non-diabetics, while T-SPOT.TB showed approximately 93% sensitivity irrespective of diabetic status (93). Overall, IGRA are considered more reliable, including in the context of DM and immune impairment, whereas TST may underestimate LTBI due to weakened immune responses and prior BCG vaccination. In a longitudinal study of T2DM patients in China, IGRA detected LTBI in 14.85% compared with 9.65% by TST at baseline. After 3 months, prevalence increased to ∼19.6% for IGRA and 21% for TST, largely attributable to the “boosting” effect (94). It should also be noted that microcirculatory disturbances in patients with diabetes may affect IFN-γ responses in IGRA, potentially influencing test performance (95). Diabetes mellitus is associated with an increased prevalence of LTBI and a higher risk of progression to active tuberculosis. IGRA tests demonstrate greater reliability and diagnostic sensitivity in diabetic patients compared with TST, which is prone to underestimation due to impaired immune responses and prior BCG vaccination. The consistently elevated rates of LTBI among individuals with diabetes underscore the need for targeted screening and preventive strategies in this population. Further research is required to clarify the role of metabolic and microcirculatory disturbances in modulating interferon-γ responses and their impact on immunodiagnostic accuracy.

### 5.6 Autoimmune diseases

Rheumatoid arthritis (RA) is one of the most common chronic autoimmune diseases in Europe and, particularly, North America, with a prevalence of 0.8–1.1%, compared with a global prevalence of 0.24% (96). Patients with RA are at an elevated risk of developing active tuberculosis in the presence of LTBI due to long-term immunosuppressive therapy and immune dysregulation. Targeted therapies used in the management of RA may contribute to neutropenia and increase susceptibility to bacterial co-infections. Several cohort and retrospective studies have demonstrated a higher prevalence of LTBI among RA patients compared with the general population, with reports of up to a fourfold higher incidence of tuberculosis. The prevalence of LTBI before initiation of anti-cytokine therapy was relatively low (7.25%) and comparable to that of the general population; however, during treatment, LTBI was identified in 21.7% of patients, indicating that individuals with RA require close monitoring, especially those receiving biologic agents (97). Moreover, RA and tuberculosis share an important common risk factor—smoking (98, 99). In recent years, the use of targeted therapies has significantly improved outcomes in rheumatologic diseases. Nevertheless, given their impact on cytokines essential for anti-tuberculosis immunity and their subsequent modulation of immune regulation, the risk of infections—including viral, bacterial, fungal, and mycobacterial—remains a challenge. In Saudi Arabia, 410 patients receiving adalimumab, etanercept, or tocilizumab were screened for LTBI using TST and IGRA. The use of these biologics was not associated with an increased risk of tuberculosis; only 0.3% of patients on adalimumab and 0.9% on etanercept converted to IGRA-positive status during therapy. However, it cannot be excluded that some of these patients had inherently reduced immune responsiveness, which may have influenced the absence of test conversion (100). Compared with TNF-α inhibitors, IL-17A inhibitors are considered less risky with respect to TB reactivation. In Turkey, a TB-endemic country, patients with psoriasis receiving secukinumab or ixekizumab for more than 12 months were screened using QuantiFERON-TB Gold In-Tube. Among 334 initially IGRA-negative patients, 10 converted to IGRA-positive during therapy. While IGRA positivity does not confirm active tuberculosis, it may serve as an indicator for closer monitoring (101). Similarly, in a Chinese cohort of 306 patients treated with IL-17A inhibitors for psoriasis, 17 out of 220 initially IGRA-negative individuals became IGRA-positive, and one case of active tuberculosis was reported. Both IGRA-negative and IGRA-positive patients demonstrated an increase in IFN-γ levels over time, suggesting that the risk of LTBI remains elevated in immunosuppressed individuals (102).

Systemic vasculitis also represents a significant autoimmune condition associated with increased TB risk. In one study, LTBI was diagnosed in 31.4% of 191 patients with systemic vasculitis using T-SPOT.TB (103). By comparison, a large multicenter study in rural China reported an LTBI prevalence of approximately 20.3% among individuals aged 15 years and older (104). These findings further support the elevated risk of tuberculosis among patients receiving immunosuppressive agents. Importantly, lymphopenia and high-dose glucocorticoid therapy were found to be associated with false-negative T-SPOT.TB results (103). Thus, when interpreting LTBI test outcomes in rheumatology patients, both the effect of therapy and the immunological state must be considered. Discrepancies between immunological tests remain a challenge. In a study of 5224 patients with rheumatic diseases, both TST and IGRA were performed prior to TNF inhibitor therapy. Positive rates for TST, QuantiFERON-TB Gold In-Tube, and T-SPOT.TB were 29, 17, and 18%, respectively, with concordance between TST and QFT-GIT of 73%, and between TST and T-SPOT of 75% (105). These findings suggest that reliance on a single test is insufficient for LTBI diagnosis in autoimmune disease patients.

Furthermore, not only can immunosuppressive therapy for autoimmune diseases increase TB risk, but anti-tuberculosis therapy itself may exacerbate or trigger autoimmune conditions. Drugs such as isoniazid and rifampicin (less commonly PAS and ceftriaxone) have been linked to lupus erythematosus, Hashimoto’s thyroiditis, multiple sclerosis, autoimmune hemolytic anemia, neutropenia, thrombocytopenia, and hemorrhaphilia caused by autoantibodies against coagulation factor XIII. The clinical risk of isoniazid-induced lupus has been estimated at approximately 1% (106–108). Moreover, interferon-γ—the cytokine central to IGRA testing—has also been implicated as a driver of autoimmunity, with enhanced production documented in predisposed individuals (109). Patients with autoimmune diseases represent a population at high risk of LTBI and progression to active tuberculosis due to underlying immune dysregulation and the use of immunosuppressive or biologic therapies. While biologics such as TNF-α inhibitors confer a particularly high risk, other agents, including IL-17A inhibitors, also warrant careful surveillance. Diagnostic challenges persist due to variability and limited concordance between TST and IGRA results, which may be further influenced by immunosuppressive therapy and patient immune status. Additionally, the bidirectional relationship between tuberculosis treatment and autoimmunity complicates management, as certain anti-TB agents may trigger or exacerbate autoimmune conditions. These findings highlight the need for combined diagnostic approaches, rigorous LTBI screening, and individualized monitoring in patients with autoimmune diseases undergoing immunosuppressive treatment.

### 5.7 Organ transplantation

Organ transplant recipients face a significantly increased risk of active tuberculosis (TB), estimated at 20–74 times higher compared to the general population. Most cases represent reactivation of latent tuberculosis infection (LTBI). According to a meta-analysis, the mean incidence of active TB after transplantation is approximately 3% (110). Interferon-gamma release assays (IGRAs) are widely applied for LTBI screening before or after transplantation. In a study by LTBI status was evaluated in 20 patients both before and after organ transplantation under immunosuppressive therapy (111). QuantiFERON-TB Gold Plus results were assessed pre- and post-transplantation. Findings revealed that in 11 patients, test results became discordant following the initiation of immunosuppressive therapy. Meanwhile, those with concordant results before and after transplantation still exhibited lower interferon-gamma responses. These data suggest that immunosuppressive therapy increases the likelihood of false-negative IGRA results. However, the small sample size and single-center design substantially limit the generalizability of these findings. Confidence intervals and effect size estimates were not reported, which constrains the interpretation of statistical robustness. Therefore, while the study provides valuable preliminary insight into the effect of immunosuppression on IGRA performance, larger multicenter investigations are required to validate these observations and clarify the diagnostic reliability of IGRAs in transplant recipients.

IGRA testing may also be useful for assessing the efficacy of preventive chemotherapy after transplantation. For example, in the study by Zeng et al. (112) kidney transplant recipients received isoniazid as preventive therapy against TB. This approach proved effective: IGRA-positive patients who received isoniazid were less likely to develop active TB compared to those who did not receive prophylaxis. Monitoring of immune status against *M. tuberculosis* before and after preventive therapy was performed using IGRA testing.

A meta-analysis of 43 studies involving 36,403 patients demonstrated that both TST and IGRA have low positive predictive value (PPV) (TST—2.13%, IGRA—1.2%) but very high negative predictive value (NPV) (IGRA—99.6%, TST—95.5%). This indicates that negative results reliably exclude the risk of progression, while positive results do not guarantee disease development. Currently, American and international guidelines (AST, IDSA, CDC, TBNET, ECDC) recommend mandatory LTBI screening for both organ donors and transplant candidates, using TST and/or IGRA (113). Organ transplantation is associated with a markedly elevated risk of TB, primarily through reactivation of latent infection. While IGRA tests are a valuable tool for pre- and post-transplant screening, their sensitivity may be compromised by immunosuppressive therapy, leading to false negatives. Preventive isoniazid therapy has proven effective in reducing TB incidence among transplant recipients. Despite the limited predictive value of positive IGRA or TST results, their high negative predictive value supports their role in reliably excluding progression to active TB. International recommendations emphasize the necessity of systematic LTBI screening for both donors and recipients to mitigate the risk of post-transplant TB.

## 6 Opportunities of artificial intelligence and machine learning

Neural networks have become an indispensable tool in solving complex diagnostic problems, gradually replacing classical machine learning approaches in areas characterized by high-dimensional data and complex spatiotemporal patterns. Their key advantage lies in their ability to automatically extract relevant features and learn complex nonlinear interactions, which are critical for such non-trivial tasks as diagnosing latent tuberculosis infection (LTBI). Modern systematic reviews note a steady increase in publications where neural network technologies are either used as standalone classifiers or as tools for generating informative representations (feature embeddings) for further analysis using traditional algorithms (114). Artificial Intelligence (AI) systems are increasingly being integrated into diagnostic platforms. For example, the combination of IGRA results, clinical risk factors, and radiographic data using neural network algorithms enhances diagnostic accuracy. AI enables risk stratification (e.g., HIV-infected patients, immunosuppressed individuals, children) by predicting the likelihood of LTBI progressing to active disease. Prognostic calculators are under development, where immunological, genetic, and epidemiological data are used to estimate an individual’s risk of reactivation. Mathematical modeling helps reproduce the dynamics of interactions between *Mycobacterium tuberculosis* and immune cells (macrophages, T lymphocytes), allowing identification of key markers associated with the transition from latent infection to active disease. Agent-based models are employed to simulate granuloma formation, bacterial distribution in tissues, and the balance between pathogen elimination and persistence. These models assist in predicting outcomes, including the immune profiles most likely to result in LTBI progression. The most successful area of application of neural network technologies in TB diagnostics remains computer vision for analyzing immunological images. In one work, a two-stage architecture based on convolutional neural networks (CNNs) was proposed for processing T-SPOT.TB assay images (115). The CNN learns from “spot-pictures” to automatically extract quantitative and spatial characteristics. These extracted features are then utilized in a final logistic model, enhancing diagnostic resolution for differentiating between active TB and LTBI compared to standard manual methods.

The development of omics technologies combined with advanced machine learning (ML) methods marks a new era in the diagnosis of latent tuberculosis infection (LTBI), enabling a transition from non-specific clinico-radiological criteria to precise molecular signatures. Our analysis demonstrated that both transcriptomic and proteomic signatures based on host immune response show great potential for distinguishing active tuberculosis (TB) from latent infection, achieving Area Under Curve (AUC) values ranging from 0.85 to 0.98. The most reproducible findings highlight interferon-regulated genes (such as GBP2, CXCL10, IFITM3), underscoring the central role of IFN-I/II-mediated responses in the pathogenesis of active TB. Successful translational pathways from broad omics discovery to short validated qRT-PCR signatures demonstrate a realistic trajectory toward clinical implementation. The integration of multi-omics data (transcriptomics, proteomics, metabolomics) through AI may lead to the creation of comprehensive “risk biomarkers” for LTBI. Personalized medicine approaches involve AI-driven selection of preventive treatment strategies according to predicted reactivation risk. On a global scale, AI facilitates the analysis of epidemiological data from different regions, accounting for geographical variability. Recently, advances in AI and bioinformatics have enabled new strategies to improve the differential diagnosis of LTBI and active TB. For instance, Gong W (2021) reports on such developments. Zhou et al. applied Immuno Score, originally used for colorectal cancer prognosis, to differentiate LTBI from TB based on cytokine profiles. Their model demonstrated high diagnostic accuracy, with 95.7% sensitivity and 92.1% specificity (42, 44). Similarly, Ndzi et al. (45) conducted an *in silico* study on HLA distribution in TB patients. IGRA-positive individuals underwent DNA genotyping, and associations were identified between specific HLA alleles/haplotypes and LTBI or progression to active TB. Based on these findings, the authors proposed a computational mapping model to predict LTBI or active TB development (45). The Cox proportional hazards model was used by Abedi et al. (116) to identify risk factors for mortality among TB patients. Male sex, TB/HIV co-infection, and cancer comorbidity were found to be major determinants of death during treatment (116). Wu et al. developed a random forest algorithm to distinguish TB from LTBI using T-SPOT.TB data (117). It is increasingly important not only to detect LTBI but also to assess the risk of progression. However, validated tools for personalized risk prediction are still lacking. Gupta et al. (118) analyzed data from > 80,000 individuals across 20 low-incidence countries (≤20/100,000 annually). They estimated 5-year cumulative TB risk among untreated LTBI carriers: 15.6% in children, 4.8% in adults, 5.0% in migrants, and 4.8% in immunocompromised individuals. The highest risk occurred within the first year after infection, gradually declining thereafter. Based on these data, the authors developed PERISKOPE-TB, a personalized risk predictor combining quantitative T-cell sensitization metrics with clinical covariates. This model may help guide preventive chemotherapy decisions and inform individualized monitoring schedules for LTBI patients in outpatient TB settings (118). Mathematical modeling has also been applied to global prevalence estimates. Houben and Dodd used regression analysis to estimate the worldwide LTBI burden at 1.7 billion individuals (∼25% of the global population) in 2014 (118). Subsequent modeling by Knight et al. estimated the prevalence of multidrug-resistant LTBI at 0.3% in the same year (119). Despite their promise, AI systems face several limitations. In many regions, especially developing countries, high-quality data for training models remain scarce. Some algorithms function as “black boxes,” limiting clinical implementation. Moreover, AI use raises concerns about patient data confidentiality. In summary, AI and machine learning are already playing an active role in TB management—from diagnostics to therapeutic development. These technologies improve diagnostic accuracy, support treatment optimization, and enable resistance prediction. However, to maximize their impact, further research, improved data accessibility, and close collaboration between scientists, clinicians, and technology developers are essential.

Key limitations of current evidence base and ML approaches revolve around improving statistical robustness and generalizability of models:

1. Reproducibility and Validation Challenges Many published models have been tested only internally or on limited external datasets. Absence of multicenter, multinational validation reduces confidence in transferring these models to new populations (e.g., HIV co-infected individuals, regions with high prevalence of nontuberculous mycobacteria). Reliable reporting standards like TRIPOD/PROBAST and public availability of frozen algorithm versions are essential to ensure reliability.
2. Data Heterogeneity and Batch Effects Variations in sample collection methods (whole blood vs. peripheral blood mononuclear cells, PBMC), platforms (Microarray vs. RNA-seq, ELISA vs. Luminex), and stimulation protocols (purified protein derivative, PPD vs. ESAT-6/CFP-10) create batch effects that ML models may misinterpret as disease-related biological signals. Additionally, dynamic range differences in protein concentrations in blood pose challenges in proteomics studies. Standardized operating procedures (SOPs) must be strictly unified, and integration methods should harmonize heterogeneous datasets.
3. Accuracy versus Interpretability Trade-offMore accurate ensemble ML models (boosting, random forests) often act as black boxes, making them difficult to interpret clinically and hindering trust among physicians. Explainable AI techniques, particularly SHAP/LIME, should be incorporated into workflows to clarify each biomarker’s contribution and justify decisions.
4. Focus on Diagnosis Rather Than PredictionMost studies focus solely on differential diagnosis at a single point in time. To achieve real prevention of LTBI progression, longitudinal cohorts and predictive models capable of forecasting the risk of LTBI progressing to active TB over time (time-dependent AUC) are needed. This requires long-term follow-up of high-risk cohorts.

The implementation of combined immune system testing models or risk prediction models based on machine learning is a multifaceted process encompassing economic, infrastructural, technological, and ethical dimensions within healthcare systems. One major hurdle lies in the substantial initial costs associated with designing and deploying machine learning-based solutions. This includes procuring hardware for computational power, specialized software, and employing highly qualified personnel to construct and sustain these models. Despite sizable upfront investments, future efficiencies may arise from reduced expenditure on redundant laboratory investigations and treatment of advanced stage diseases. Creating an environment conducive to implementing machine learning models mandates dependable digital storage systems for patient data, servers, and high-performance computing resources. At the same time, healthcare institutions must modernize their IT infrastructure by integrating existing medical informatics systems with innovative technologies. Security measures play a decisive role, as ensuring the confidentiality of medical records is essential. The effectiveness of machine learning models substantially depends on the volume and quality of input data. If datasets are inadequate or contain multiple errors and missing records, resultant models will perform inefficiently. It is thus imperative to establish continuous processes for updating and monitoring data quality, incorporating regular assessments to identify inconsistencies and resolve issues relating to data completeness.

The use of artificial intelligence in medicine raises important ethical questions concerning patient rights and data privacy. Critical concerns encompass obtaining informed consent for data processing, fairly distributing benefits arising from new technologies, and avoiding misuse in decision-making influenced by AI. Patients must retain the right to refuse experimental diagnostic methods based on machine learning, even if this leads to increased financial burdens for healthcare providers. Furthermore, a notable issue is the presence of bias in machine learning models due to underrepresentation of specific ethnic or socioeconomic groups in training datasets. Such disparities lead to inaccurate diagnoses or inappropriate recommendations for targeted segments of the population. Routinely conduct audits and refresh datasets to minimize incorrect data points. Introduce centralized data governance frameworks to track data provenance and enforce uniform quality standards. Enhance employee proficiency in handling large datasets and applying machine learning tools.

Invest in infrastructure capable of storing and processing massive volumes of data. Facilitate ongoing communication channels between data analysts, clinicians, and software developers to prevent technical breakdowns and guard against breaches of confidentiality. Such measures will elevate the efficiency of combining immune system testing and risk prediction models built on machine learning, minimizing risks while maximizing benefits for patients and the entire medical community.

## 7 Conclusion

The incidence of tuberculosis (TB) in the Russian Federation has decreased over the past 13 years and, as of 2024, is 26.49 cases per 100,000 population. This represents a positive trend, especially considering that during 2021–2023 the global TB incidence increased, partly due to the COVID-19 pandemic. Despite the decline in TB morbidity in Russia, the true prevalence of latent TB infection (LTBI), which serves as a reservoir for active disease, remains unknown. Given the complex mechanisms of mycobacterial persistence in the human body, timely detection of LTBI in risk groups represents an important strategy for reducing TB incidence. In certain populations, LTBI prevalence is higher compared with the general healthy population. Such risk groups include individuals with diabetes mellitus; autoimmune diseases; organ transplant recipients; patients undergoing hemodialysis or immunosuppressive therapy; people living with HIV; and socially vulnerable groups, among others. A particularly important category is represented by close contacts of patients with pulmonary TB (both adults and children, including adolescents under 18 years), as well as healthcare workers. According to various data, medical staff, especially those directly involved in the treatment of TB patients, are at greater risk of LTBI exposure, which underscores the need for a tailored surveillance strategy. Moreover, the duration and frequency of contact with an infectious TB patient increase the likelihood of acquiring LTBI, a factor that should be considered in risk analysis. Currently, several immunological tests are used in Russia, including the tuberculin skin test (Mantoux test), QuantiFERON-TB Plus, T-SPOT.TB, and Diaskintest^®^. Despite the introduction of new diagnostic tools, LTBI detection remains a challenge both in Russia and worldwide. This is due to the following limitations: the inability of immunological tests to differentiate LTBI from active TB; limited accuracy in children recently vaccinated or revaccinated with BCG; dependency of test results on immune status in HIV-infected individuals, patients with congenital or acquired immunodeficiencies, diabetes, immunosuppressive therapy, or hemodialysis; as well as age, disease extent (minimal or advanced TB), and individual immune reactivity. These factors can result in either underdiagnosis or overdiagnosis of LTBI due to false-negative or false-positive results, leading to delayed or unnecessary preventive chemotherapy (120, 121). Below is a summary table presenting data on the effectiveness of various immunological tests for the diagnosis of latent tuberculosis infection (LTBI) and/or differentiation of LTBI from active tuberculosis. In Table 5, we have consolidated key quantitative findings—such as study population, diagnostic method (TST, IGRA, or combined), sample size, and LTBI prevalence. The table provides key performance indicators (sensitivity, specificity, AUC, or other metrics) extracted from available meta-analyses and systematic reviews.

**TABLE 5:** The key quantitative findings—such as study population, diagnostic method (TST, IGRA, or combined), sample size, and LTBI prevalence.

Immunologic test	Population/context	Sensitivity (%)	Specificity (%)	References
Tuberculin Skin Test (TST)	Healthcare workers in endemic area (South Africa)	93	57	(122)
IGRA (QuantiFERON-TB Gold In-Tube)	Same population	80	96	(122)
TST (pre-transplantation)	Organ transplant candidates	46	86	(123)
IGRA (pre-transplantation)	Same population	58	89	(123)
Cytokine IL-2	ATB vs. LTBI, meta-analysis of 14 studies	87	61	(124)
Cytokine IP-10	ATB vs. LTBI, meta-analysis	77	73	(124)
TBST (antigen-based skin test)	TB infection diagnosis (various groups)	77,9	80,3	(125)
IGRA (LIOFeron^®^TB/LTBI in children)	Children (LTBI screening)	100		(126)
Diaskintest	Children (LTBI screening)	91–100	88	(27, 122, 123, 127)

Another important issue is the occurrence of discordant results between different immunological tests in the same individual, explained both by test mechanisms and by the specific features of the patient’s immune system. This may complicate clinical decision-making regarding the presence or absence of LTBI in a given individual. Taken together, the data highlight the relevance of studying LTBI prevalence in various risk groups to develop evidence-based recommendations for detection and preventive treatment as a strategy to reduce the incidence of active TB. However, given the limitations of current diagnostic systems in terms of sensitivity and specificity, a conditional “combined approach”—i.e., the concurrent use of multiple immunological tests in combination with laboratory and instrumental methods in the same individuals—appears justified. Developing a systematic strategy for LTBI detection in risk groups may facilitate early identification of infection and play an important role in preventing progression to active TB.

## Footnotes

1
http://www.prisma-statement.org

## References

[1] Starshinova A Kudryavtsev I Rubinstein A Malkova A Dovgaluk I Kudlay D. Tuberculosis and COVID-19 dually affect human Th17 cell immune response. *Biomedicines.* (2023) 11:2123. 10.3390/biomedicines11082123 37626620 PMC10452633

[2] Kudryavtsev I Starshinova A Rubinstein A Kulpina A Ling H Zhuang M The role of the immune response in developing tuberculosis infection: from latent infection to active tuberculosis. *Front Tuberc.* (2024) 2:1438406. 10.3389/ftubr.2024.1438406 PMC13494988 42638763

[3] World Health Organization. *Global Tuberculosis Report 2024.* Geneva: World Health Organization (2024). Licence: CC BY-NC-SA 3.0 IGO.

[4] Migliori GB Thong PM Akkerman O Alffenaar JW Álvarez-Navascués F Assao-Neino MM Worldwide effects of coronavirus disease pandemic on tuberculosis services, January-April 2020. *Emerg Infect Dis.* (2020) 26:2709–12. 10.3201/eid2611.203163 32917293 PMC7588533

[5] World Health Organization. *Global Tuberculosis Report 2022.* Geneva: World Health Organization (2022). Licence: cc bY-Nc-sa 3.0 iGo.

[6] О состоянии санитарно-эпидемиологического благополучия населения в Российской Федерации в 2024 году: Государственный доклад. М.: Федеральная служба по надзору в сфере защиты прав потребителей и благополучия человека, (2025). 424 c p.

[7] Cohen A Mathiasen VD Schön T Wejse C. The global prevalence of latent tuberculosis: a systematic review and meta-analysis. *Eur Respir J.* (2019) 54:1900655. 10.1183/13993003.00655-2019 31221810

[8] World Health Organization. *Latent Tuberculosis Infection: Updated and Consolidated Guidelines for Programmatic Management.* Geneva: World Health Organization (2018). 30277688

[9] Zellweger JP Sotgiu G Corradi M Durando P. The diagnosis of latent tuberculosis infection (LTBI): currently available tests, future developments, and perspectives to eliminate tuberculosis (TB). *Med Lav.* (2020) 111:170–83. 10.23749/mdl.v111i3.9983 32624559 PMC7809945

[10] Getahun H Matteelli A Abubakar I Aziz MA Baddeley A Barreira D Management of latent *Mycobacterium tuberculosis* infection: WHO guidelines for low tuberculosis burden countries. *Eur Respir J.* (2015) 46:1563–76. 10.1183/13993003.01245-2015 26405286 PMC4664608

[11] Vishnevsky BI Yablonsky PK. Persistence of *Mycobacterium tuberculosis* - the basis of latent tuberculosis (literature review). *Med Alliance.* (2020) 8:14–20. 10.36422/23076348-2020-8-2-14-20

[12] Slogotskaya LV Lovacheva OV Klevno NI. Stages of tuberculosis infection, what’s new? (literature review). *Tuberk Lung Dis.* (2025) l103:93–101. 10.58838/2075-1230-2025-103-2-93-101

[13] Knight GM McQuaid CF Dodd PJ Houben RMGJ. Global burden of latent multidrug-resistant tuberculosis: trends and estimates based on mathematical modelling. *Lancet Infect Dis*. 2019 19:903–912. 10.1016/S1473-3099(19)30307-X Epub 2019 Jul 4. *Erratum in: Lancet Infect Dis.* (2020) 20:e50. doi: 10.1016/S1473-3099(20)30087-6 31281059 PMC6656782

[14] Buron V Banaei N. Inflated gamma interferon response with QuantiFERON-TB gold plus using the automated liaison XL analyzer: a testing algorithm to mitigate false-positive results in low-incidence settings. *J Clin Microbiol*. 2023;61:e0029523. 10.1128/jcm.00295-23 Epub 2023 May 17. *Erratum in: J Clin Microbiol.* (2023) 61:e0106723. doi: 10.1128/jcm.01067-23 37195172 PMC10281139

[15] World Health Organization. *WHO Consolidated Guidelines on Tuberculosis. Module 5: Management of Tuberculosis in Children and Adolescents.* Geneva: World Health Organization (2022). 35404556

[16] Starshinova A. Difficulties in diagnosing tuberculosis infection in childhood. *Lancet Infect Dis.* (2024) 25:140–1. 10.1016/S1473-3099(24)00569-3 39312917

[17] Ou X Liu E Rashid F Pei S Zhang G Anthony R Screening and preclinical assessment of novel *Mycobacterium tuberculosis* recombinant antigens based tuberculin skin testing. *Front Immunol.* (2025) 16:1498448. 10.3389/fimmu.2025.1498448 40124357 PMC11925772

[18] Serge Diagbouga P Djibougou AD Pease C Alcaide A Berthoux A Bruiners N Preliminary performance of the VIDAS TB-IGRA as an aid in the diagnosis of individuals infected with *Mycobacterium tuberculosis*. *J Clin Microbiol.* (2025) 63:e0164124. 10.1128/jcm.01641-24 40366166 PMC12153259

[19] Budritsky AM Levyankova AL Volovik DV Shushman MA. Comparative study of the diagnostic capabilities of QuantiFERON-TB Gold Plus and other types of tests for tuberculosis infection. *Clin Infectol Parasitol.* (2024) 13:447–53. 10.34883/PI.2024.13.4.031

[20] Ghanaie RM Karimi A Azimi L James S Nasehi M Mishkar AP Diagnosis of latent tuberculosis infection among pediatric household contacts of Iranian tuberculosis cases using tuberculin skin test, IFN-γ release assay and IFN-γ-induced protein-10. *BMC Pediatr.* (2021) 21:76. 10.1186/s12887-021-02524-3 33573613 PMC7877026

[21] Godoy P. Guidelines on controlling latent tuberculosis infection to support tuberculosis elimination. *Rev Esp Sanid Penit.* (2021) 23:28–36. 10.18176/resp.00028 33847703 PMC8278168

[22] Zhang Y Zhou G Shi W Shi W Hu M Kong D Comparing the diagnostic performance of QuantiFERON-TB Gold Plus with QFT-GIT, T-SPOT.TB and TST: a systematic review and meta-analysis. *BMC Infect Dis.* (2023) 23:40. 10.1186/s12879-023-08008-2 36670347 PMC9862551

[23] Pagnoncelli M Arosio M Genovesi A Napolitano G Farina C. Performance of the T-SPOT.TB test in patients with indeterminate QuantiFERON-TB Gold Plus results: proposal for an algorithm for the diagnosis of Latent Tuberculosis Infection. *Infez Med.* (2024) 32:525–31. 10.53854/liim-3204-11 39660151 PMC11627487

[24] World Health Organization. *Rapid Communication: TB Antigen-Based Skin Tests for the Diagnosis of TB Infection.* Geneva: World Health Organization (2022). Licence: CC BY-NC-SA 3.0 IGO.

[25] Hamada Y Cirillo DM Matteelli A Penn-Nicholson A Rangaka MX Ruhwald M. Tests for tuberculosis infection: landscape analysis. *Eur Respir J.* (2021) 58:2100167. 10.1183/13993003.00167-2021 33875495

[26] Gordeev AA Plekhanova MA Smerdin SV Tkachuk AP Manuylov VA Kudryashova AM comparative study of six immunological tests for the diagnosis of tuberculosis in different patient groups. *Tuberc Lung Dis.* (2025) 103:94–102. 10.58838/2075-1230-2025-103-3-94-102

[27] Starshinova A Zhuravlev V Dovgaluk I Panteleev A Manina V Zinchenko U A comparison of intradermal test with recombinant tuberculosis allergen (diaskintest) with other immunologic tests in the diagnosis of tuberculosis infection. *Int J Mycobacteriol.* (2018) 7:32–9. 10.4103/ijmy.ijmy_17_18 29516883

[28] Krutikov M Faust L Nikolayevskyy V Hamada Y Gupta RK Cirillo D The diagnostic performance of novel skin-based in-vivo tests for tuberculosis infection compared with purified protein derivative tuberculin skin tests and blood-based in vitro interferon-γ release assays: a systematic review and meta-analysis. *Lancet Infect Dis*. 2022;22:250–64. 10.1016/S1473-3099(21)00261-9 Epub 2021 Oct 1. *Erratum in: Lancet Infect Dis.* (2022) 22:e41. doi: 10.1016/S1473-3099(21)00651-4 34606768

[29] Starshinova A Dovgalyk I Malkova A Zinchenko Y Pavlova M Belyaeva E Recombinant tuberculosis allergen (Diaskintest^®^) in tuberculosis diagnostic in Russia (meta-analysis). *Int J Mycobacteriol.* (2020) 9:335–46. 10.4103/ijmy.ijmy_131_20 33323648

[30] Vasilyeva IA Aksenova VA Kazakov AV Kiseleva YY Maryandyshev AO Dolzhenko EN Evaluation of the specificity of an intradermal test with recombinant tuberculosis allergen in bacillus Calmette-Guérin-vaccinated healthy volunteers. *Front Med.* (2023) 10:104246. 10.3389/fmed.2023.1042461 36936243 PMC10014891

[31] Chin KL Anibarro L Sarmiento ME Acosta A. Challenges and the way forward in diagnosis and treatment of tuberculosis infection. *Trop Med Infect Dis.* (2023) 8:89. 10.3390/tropicalmed8020089 36828505 PMC9960903

[32] Latorre I Mínguez S Carreras F IP-10 release assays as biomarkers for active tuberculosis, latent infection and response to treatment. *Front Immunol.* (2023) 14:1123451. 10.3389/fimmu.2023

[33] Izumida M Jobe H Coker EG Barry A Rashid M Manneh IL HBHA induces IL-10 from CD4+ T cells in patients with active tuberculosis but IFN-g and IL-17 from individuals with *Mycobacterium tuberculosis* infection. *Front Immunol.* (2024) 15:1422700. 10.3389/fimmu.2024.1422700 39257584 PMC11384583

[34] Petruccioli E Scriba TJ Petrone L Correlates of tuberculosis risk: predictive biomarkers for progression to active tuberculosis. *Eur Respir J.* (2020) 55:1902469. 10.1183/13993003.02469-2019 PMC5898936 27836953

[35] Duffy FJ Thompson EG Scriba TJ. Immunological biomarkers of tuberculosis. *Nat Rev Immunol.* (2021) 21:162–76. 10.1038/s41577-020-00448-5 21475309

[36] Andersen P Scriba TJ. Moving tuberculosis vaccines from theory to practice. *Nat Rev Immunol.* (2022) 22:588–601. 10.1038/s41577-022-00790-y 31114037

[37] Nemes E Rozot V Geldenhuys H Differential recognition of *Mycobacterium tuberculosis*-specific epitopes as markers of latent infection stability and risk of progression. *Clin Infect Dis.* (2024) 78:456–66. 10.1093/cid/ciad567 38118015 PMC10732560

[38] Abubakar I Drobniewski F Southern J Sitch AJ Jackson C Lipman M Prognostic value of interferon-γ release assays and tuberculin skin test in predicting the development of active tuberculosis (UK PREDICT TB): a prospective cohort study. *Lancet Infect Dis.* (2018) 18:1077–87. 10.1016/S1473-3099(18)30355-4 30174209 PMC6192014

[39] Fletcher HA Nemes E. Editorial: biomarkers of tuberculosis disease progression and treatment response. *Front Tuberc.* (2025) 5:1623373. 10.3389/ftubr.2025.1623373

[40] Luo Q Zhu B Zhang L Liu Z Wu Q Chen M Plasma GM-CSF, CXCL10 and IL-1Ra as predictive biomarkers for the progression from latent tuberculosis infection to active tuberculosis. *Front Immunol.* (2024) 15:1371817. 10.3389/fimmu.2024.1371817

[41] Flynn JL Chan J. Immune cell interactions in tuberculosis. *Cell.* (2022) 185:4682–702. 10.1016/j.cell.2022.10.025 36493751 PMC12162144

[42] Gong W Wu X. Differential diagnosis of latent tuberculosis infection and active tuberculosis: a key to a successful tuberculosis control strategy. *Front Microbiol.* (2021) 12:745592. 10.3389/fmicb.2021.745592 34745048 PMC8570039

[43] Garlant HN Ellappan K Hewitt M Perumal P Pekeleke S Wand N Evaluation of host protein biomarkers by ELISA from whole lysed peripheral blood for development of diagnostic tests for active tuberculosis. *Front Immunol.* (2022) 13:854327. 10.3389/fimmu.2022.854327 35720382 PMC9205408

[44] Zhou Y Du J Hou HY Lu YF Yu J Mao LY Application of immunoscore model for the differentiation between active tuberculosis and latent tuberculosis infection as well as monitoring anti-tuberculosis therapy. *Front Cell Infect Microbiol.* (2017) 7:457. 10.3389/fcimb.2017.00457 29164066 PMC5670161

[45] Ndzi EN Nkenfou CN Pefura EWY Mekue LCM Guiedem E Nguefeu CN Tuberculosis diagnosis: algorithm that may discriminate latent from active tuberculosis. *Heliyon.* (2019) 5:e02559. 10.1016/j.heliyon.2019.e02559 31692671 PMC6806400

[46] Petruccioli E Vanini V Chiacchio T CD27 expression as a tool to discriminate between active and latent tuberculosis. *J Infect Dis.* (2015) 211:936–44. 10.1093/infdis/jiu574 26253021

[47] Ai JW Ruan QL Liu QH Zhang WH. Updates on the risk factors for latent tuberculosis reactivation and their managements. *Emerg Microbes Infect.* (2023) 12:e2274931. 10.1080/22221751.2023.2274931 PMC4777925 26839146

[48] Reichler MR Hirsch C Yuan Y Khan A Dorman SE Schluger N Predictive value of TNF-α, IFN-γ, and IL-10 for tuberculosis among recently exposed contacts in the United States and Canada. *BMC Infect Dis.* (2020) 20:553. 10.1186/s12879-020-05185-2 32736606 PMC7394686

[49] Suliman S Thompson EG Sutherland J Weiner J Ota MOC Shankar S Four-gene Pan-African blood signature predicts progression to tuberculosis. *Am J Respir Crit Care Med.* (2018) 197:1198–208. 10.1164/rccm.201711-2340OC 29624071 PMC6019933

[50] Drain PK Bajema KL Dowdy D Dheda K Naidoo K Schumacher SG Incipient and subclinical tuberculosis: a clinical review of early stages and progression of infection. *Clin Microbiol Rev.* (2018) 31:e21–18. 10.1128/CMR.00021-18 30021818 PMC6148193

[51] Scriba TJ Penn-Nicholson A Shankar S Hraha T Thompson EG Sterling D Sequential inflammatory processes define human progression from *M. tuberculosis* infection to tuberculosis disease. *PLoS Pathog.* (2017) 13:e1006687. 10.1371/journal.ppat.1006687 29145483 PMC5689825

[52] Vasiliu A Martinez L Gupta RK Hamada Y Ness T Kay A Tuberculosis prevention: current strategies and future directions. *Clin Microbiol Infect.* (2024) 30:1123–30. 10.1016/j.cmi.2023.10.023 37918510 PMC11524220

[53] Santos JA Duarte R Nunes C. Host factors associated to false negative and indeterminate results in an interferon-γ release assay in patients with active tuberculosis. *Pulmonology.* (2020) 26:353–62. 10.1016/j.pulmoe.2019.11.001 31843341

[54] Zheng H Xiao J Li F Chen H Li D Wang Y Interferon-gamma release assay for screening of tuberculosis infection in children. *BMC Infect Dis.* (2023) 23:873. 10.1186/s12879-023-08871-z 38093183 PMC10717111

[55] Li Q Ren W Yuan J Guo H Shang Y Wang W Significant difference in Th1/Th2 paradigm induced by tuberculosis-specific antigens between IGRA-positive and IGRA-negative patients. *Front Immunol.* (2022) 13:904308. 10.3389/fimmu.2022.904308 36119060 PMC9471257

[56] Godoy S Alsedà M Parrón I Millet JP Caylà JA Follia N Exposure time to a tuberculosis index case as a marker of infection in immigrant populations. *Pathogens.* (2025) 14:175. 10.3390/pathogens14020175 40005550 PMC11858108

[57] Choi Y Park SJ An HS Kim HM Yoo JY Pyo SW Epidemiological analysis of tuberculosis transmission, risk factors, and subclinical tuberculosis management in a High School Outbreak, South Korea. *Open Forum Infect Dis.* (2025) 12:ofaf452. 10.1093/ofid/ofaf452 40809392 PMC12345623

[58] Reichler MR Khan A Yuan Y Chen B McAuley J Mangura B Duration of exposure among close contacts of patients with infectious tuberculosis and risk of latent tuberculosis infection. *Clin Infect Dis.* (2020) 71:1627–34. 10.1093/cid/ciz1044 32044987 PMC8851607

[59] Sousa S Rocha D Silva JC Ribeiro AI Gonçalves G Almeida Á Comparing the cost-effectiveness of two screening strategies for latent tuberculosis infection in Portugal. *Pulmonology.* (2021) 27:493–9. 10.1016/j.pulmoe.2021.04.002 34053903

[60] Bogorodskaya EM Slogotskaya LV Shamuratova LF Sevostyanova TA. Screening for tuberculosis infection in children and adolescents using two intradermal tests: with tuberculin and recombinant tuberculosis allergen (ESAT-6/CFP-10) in Moscow in 2021. *Tuberc Lung Dis.* (2022) 100:29–38. 10.21292/2075-1230-2022-100-11-29-38

[61] Surve S Bhor V Gounder V Munne K Begum S Naukariya K Management implications of latent TB among under-five children at risk: insights from a community study in Mumbai, India. *Pediatr Pulmonol.* (2025) 60:e27336. 10.1002/ppul.27336 39422171

[62] Starshinova AA Pavlova MV Dovgalyuk IF Yakunova OA. Diagnostic capabilities of modern immunological tests in determining the activity of tuberculosis infection in children. *Tuberc Lung Dis.* (2012) 89:40–3.

[63] Starshinova AA Panteleev AM Manina VV Istomina EV Afonin DN Zhuravlev VY. Capabilities of various immunological tests in the diagnosis of tuberculosis in patients with HIV infection. *Tuberc Lung Dis.* (2016) 94:14–22.

[64] Lin WC Lin HH Lee SS Sy CL Wu KS Chen JK Prevalence of latent tuberculosis infection in persons with and without human immunodeficiency virus infection using two interferon-gamma release assays and tuberculin skin test in a low human immunodeficiency virus prevalence, intermediate tuberculosis-burden country. *J Microbiol Immunol Infect.* (2016) 49:729–36. 10.1016/j.jmii.2014.08.010 25442858

[65] Starshinova AA Zinchenko YS Istomina EV Basantsova NY Filatov MV Belyaeva EN Diagnosis of latent tuberculosis infection in institutions of various profiles and the formation of a risk group for tuberculosis. *BIOpreparations.* (2019) 2019:33–9. Available online at: https://www.biopreparations.ru/jour/article/view/244/227

[66] Sinitsyn MV Bogorodskaya EM Ayusheeva LB Belilovsky EM. Latent tuberculosis infection among HIV-infected individuals in Moscow. *Tuberc Soc Signific Dis.* (2017) 2:42–4.

[67] Cattamanchi A Smith R Steingart KR Metcalfe JZ Date A Coleman C Interferon-gamma release assays for the diagnosis of latent tuberculosis infection in HIV-infected individuals: a systematic review and meta-analysis. *J Acquir Immune Defic Syndr.* (1999) 56:230–8. 10.1097/QAI.0b013e31820b07ab 21239993 PMC3383328

[68] Borodulina EA Kudlai DA Kuznetsova AN Gladunova EP Kalashnikova EV. Use of the ELISPOT technological platform in the diagnosis of tuberculosis infection in patients with HIV infection. *Immunology.* (2021) 42:395–402. 10.33029/0206-4952-2021-42-4-395

[69] Runels T Ragan EJ Ventura AS Winter MR White LF Horsburgh CR Testing and treatment for latent tuberculosis infection in people living with HIV and substance dependence: a prospective cohort study. *BMJ Open.* (2022) 12:e058751. 10.1136/bmjopen-2021-058751 35273063 PMC8915380

[70] White HA Baggaley RF Okhai H Patel H Stephenson I Bodimeade C The impact, effectiveness and outcomes of targeted screening thresholds for programmatic latent tuberculosis infection testing in HIV. *AIDS.* (2022) 36:2035–44. 10.1097/QAD.0000000000003364 35983827 PMC9612707

[71] Pipitò L Ricci, Maggi P De Socio GV Pellicano GF Trizzino M Screening for latent tuberculosis infection in people living with HIV: TUBHIVIT project, a multicenter Italian study. *Viruses.* (2024) 16:777. 10.3390/v16050777 38793658 PMC11125621

[72] Petruccioli E Chiacchio T Navarra A Vanini V Cuzzi G Cimaglia C Effect of HIV-infection on QuantiFERON-plus accuracy in patients with active tuberculosis and latent infection. *J Infect.* (2020) 80:536–46. 10.1016/j.jinf.2020.02.009 32097688 PMC8862140

[73] Starshinova AA Dovgalyuk IF Yablonskiy PK. Immunodiagnostics of tuberculosis: 10-year experience of using immunological tests in Russia. *Tuberc Lung Dis.* (2019) 97:58–65. 10.21292/2075-1230-2019-97-5-58-65

[74] Bogorodskaya EM Mokhireva LV Musatkina NV. Prevalence of latent tuberculosis infection among workers of healthcare organizations. *Tuberc Soc Signific Dis.* (2023) 11:4–11. 10.54921/2413-0346-2023-11-1-4-11

[75] Qader GQ Seddiq MK Rashidi KM Manzoor L Hamim A Akhgar MH Prevalence of latent tuberculosis infection among health workers in Afghanistan: a cross-sectional study. *PLoS One.* (2021) 16:e0252307. 10.1371/journal.pone.0252307 34061873 PMC8168887

[76] Apriani L McAllister S Sharples K Aini IN Nurhasanah H Ratnaningsih DF Tuberculin skin test and Interferon-gamma release assay agreement, and associated factors with latent tuberculosis infection, in medical and nursing students in Bandung, Indonesia. *PLoS One.* (2024) 19:e0299874. 10.1371/journal.pone.0299874 38498488 PMC10947906

[77] Azeredo ACV Holler SR de Almeida EGC Cionek OAGD Loureiro MM Freitas AA Tuberculosis in health care workers and the impact of implementation of hospital infection-control measures. *Workplace Health Saf.* (2020) 68:519–25. 10.1177/2165079920919133 32502371

[78] Meregildo-Rodriguez, Yuptón-Chávez V Asmat-Rubio MG Vásquez-Tirado GA. Latent tuberculosis infection (LTBI) in health-care workers: a cross-sectional study at a northern Peruvian hospital. *Front Med (Lausanne).* (2023) 10:1295299. 10.3389/fmed.2023.1295299 38098842 PMC10720426

[79] Rizzi A Nucera E Mazzucco W Palumbo P Staiti D Moscato U An interoperable web-based platform to support health surveillance against latent tuberculosis infection in health care workers and students: the evolution of CROSSWORD study protocol. *PLoS One.* (2025) 20:e0319568. 10.1371/journal.pone.0319568 40132007 PMC11936157

[80] Istomina EV Zinchenko YS Belyaeva EN Basantzova NY Starshinova AA. Early detection of tuberculosis infection in employee s of tuberculosis facilities and general in-patient clinics. *Tuberc Soc Signific Dis.* (2018) 4:4–9.

[81] Istomina EV. Diagnosis of latent tuberculosis infection in employees of an anti-tuberculosis institution. *Med Alliance.* (2015) 2:47–55.

[82] Timofeev RM Marchenko AN Pirogova ND Kalashnikov AA. Analysis of tuberculosis incidence among employees of forensic medicine bureau in tyumen region in 2003-2022. *Tuberc Lung Dis.* (2024) 102:20–5. 10.58838/2075-1230-2024-102-1-20-25

[83] Bobokhodjaev I Abdurakhimov AA Bobokhodjaev FO Pirov KI. The level of latent tuberculosis infection and the experience of organizing a set of preventive measures during repeated contacts of employees of an anti-tuberculosis institution of the Republic of Tajikistan. *Bull Cent Res Inst Tuberc.* (2024) 3:32–41. 10.57014/2587-6678-2024-8-3-32-41

[84] Uddin MKM Islam A Jabin MS Alam T Khair S Ferdous J Comparative evaluation of diagnostic performance: standard E TB Feron ELISA vs QuantiFERON-TB Gold Plus for latent tuberculosis infection detection in diverse risk groups in Bangladesh. *Infect Drug Resist.* (2024) 17:3925–32. 10.2147/IDR.S475424 39280729 PMC11397246

[85] Alemu A Bitew ZW Diriba G Seid G Moga S Abdella S The prevalence of latent tuberculosis infection in patients with chronic kidney disease: a systematic review and meta-analysis. *Heliyon.* (2023) 9:e17181. 10.1016/j.heliyon.2023.e17181 37484241 PMC10361307

[86] Hayuk P Boongird S Pornsuriyasak P Bruminhent J. Interferon gamma release assays for diagnosis of latent TB infection in chronic kidney diseases and dialysis patients. *Front Cell Infect Microbiol* (2022) 12:1046373. 10.3389/fcimb.2022.1046373 36452296 PMC9701719

[87] Ogawa Y Harada M Hashimoto K Kamijo Y. Prevalence of latent tuberculosis infection and its risk factors in Japanese hemodialysis patients. *Clin Exp Nephrol.* (2021) 25:1255–65. 10.1007/s10157-021-02093-w 34129132

[88] Xia Y Fan Q Zhang J Jiang L Huang X Xiong Z Risk factors and prognosis for latent tuberculosis infection in dialysis patients: a retrospective cohort study at a single tertiary care center. *Semin Dial.* (2024) 37:59–64. 10.1111/sdi.13150 36823755

[89] Zhou G Guo X Cai S Zhang Y Zhou Y Long R Diabetes mellitus and latent tuberculosis infection: an updated meta-analysis and systematic review. *BMC Infect Dis.* (2023) 23:770. 10.1186/s12879-023-08775-y 37940866 PMC10631079

[90] Magee MJ Salindri AD Kornfeld H Singhal A. Reduced prevalence of latent tuberculosis infection in diabetes patients using metformin and statins. *Eur Respir J.* (2019) 53:1801695. 10.1183/13993003.01695-2018 30523163 PMC6709848

[91] Chang A Wu CZ Lin JD Lee CN Tsai KY Wu PH Prevalence and risk factors for latent tuberculosis among diabetes patients in Taiwan: a cross-sectional study. *J Infect Dev Ctries.* (2022) 16:644–9. 10.3855/jidc.15839 35544626

[92] Abdul-Ghani R Al-Awadi A Al-Aghbari N Al-Mikhlafy AA Abdulmoghni SS Al-Dobai SS Latent tuberculosis infection and diagnostic performance of the tuberculin skin test among type 2 diabetics in Sana’a city, Yemen. *BMC Infect Dis.* (2024) 24:1005. 10.1186/s12879-024-09931-8 39300351 PMC11411833

[93] Walsh MC Camerlin AJ Miles R Pino P Martinez P Mora-Guzmán F The sensitivity of interferon-gamma release assays is not compromised in tuberculosis patients with diabetes. *Int J Tuberc Lung Dis* (2011) 15:179–84,i–iii. 21219678 PMC3085021

[94] He Y Cao X Guo T He Y Du Y Zhang H Serial testing of latent tuberculosis infection in patients with diabetes mellitus using interferon-gamma release assay, tuberculin skin test, and creation tuberculin skin test. *Front Public Health.* (2022) 10:1025550. 10.3389/fpubh.2022.1025550 36530654 PMC9754324

[95] Churilov LP Normatov MG Ling H Zhuang M Kudlay D Starshinova A. Autoimmune diseases and molecular mimicry in tuberculosis. *Biology.* (2024) 13:1083. 10.3390/biology13121083 39765749 PMC11673594

[96] Chauhan K Jandu JS Brent LH Al-Dhahir MA. Rheumatoid arthritis 2023. In: *StatPearls.* Treasure Island (FL): StatPearls Publishing (2025). 28723028

[97] Malinová J Hájková M Hatalová A Šteňová E. Risk of latent tuberculosis in the cohort of patients with rheumatoid arthritis in Slovakia. *Epidemiol Mikrobiol Imunol.* (2021) 70:83–90. 34412483

[98] Hutchinson D Shepstone L Moots R Lear JT Lynch MP. Heavy cigarette smoking is strongly associated with rheumatoid arthritis (RA), particularly in patients without a family history of RA. *Ann Rheum Dis.* (2001) 60:223–7. 10.1136/ard.60.3.223 11171682 PMC1753588

[99] Feldman C Theron AJ Cholo MC Anderson R. Cigarette smoking as a risk factor for tuberculosis in adults: epidemiology and aspects of disease pathogenesis. *Pathogens.* (2024) 13:151. 10.3390/pathogens13020151 38392889 PMC10892798

[100] Boqaeid A Layqah L Alonazy A Althobaiti M Almahlawi AZ Al-Roqy A The risk of tuberculosis infection in Saudi patients receiving adalimumab, etanercept, and tocilizumab therapy. *J Infect Public Health.* (2024) 17:1134–41. 10.1016/j.jiph.2024.04.016 38728834

[101] Erbağcı E Koç Yıldırım S Hapa FA. Evaluation of serial QuantiFERON-TB Gold in tube test results and tuberculosis infection status in patients with psoriasis receiving anti-IL-17 treatment (secukinumab and ixekizumab): Real-world data from a tuberculosis-endemic country. *Australas J Dermatol.* (2024) 65:567–75. 10.1111/ajd.14340 38946637

[102] Hu K Liu Y Sha Y Zhang M Jian L Duan Y Safety of interleukin-17A inhibitors in 306 patients with psoriasis with or without latent tuberculosis: a dual-centre retrospective study in China. *Clin Exp Dermatol.* (2025) 50:1107–15. 10.1093/ced/llae549 39918838

[103] Zhong J Li Y Chen Y Shi X Zhou B Ruan G Systemic vasculitis with latent tuberculosis infection and associated factors: a cross-sectional multicenter study. *Clin Rheumatol.* (2025) 44:1269–77. 10.1007/s10067-024-07279-7 39838164 PMC11865159

[104] Gao L Lu W Bai L Wang X Xu J Catanzaro A Latent tuberculosis infection in rural China: baseline results of a population-based, multicentre, prospective cohort study. *Lancet Infect Dis.* (2015) 15:310–9. 10.1016/S1473-3099(14)71085-0 25681063

[105] Pyo J Cho SK Kim D Sung YK. Systemic review: agreement between the latent tuberculosis screening tests among patients with rheumatic diseases. *Korean J Intern Med.* (2018) 33:1241–51. 10.3904/kjim.2016.222 29277097 PMC6234384

[106] Cerqueira A Seco T Paiva D Martins H Cotter J. Isoniazid-induced lupus: when the cure can be lethal. *Cureus.* (2020) 12:e7311. 10.7759/cureus.7311 32313752 PMC7164554

[107] Sveroni D Stefos A Rigopoulou EI Dalekos GN. Rifampicin: not always an innocent drug. *BMJ Case Rep.* (2018) 11:e227356. 10.1136/bcr-2018-227356 30567221 PMC6301475

[108] Plas M Kampschreur LM Kroes JA Porcelijn L Bethlehem C. Ceftriaxone-induced thrombocytopenia during tuberculosis treatment: a case report. *Eur J Hosp Pharm.* (2025) 32:385–7. 10.1136/ejhpharm-2024-004165 39332895

[109] Liu W Zhang S Wang JIFN. -γ, should not be ignored in SLE. *Front Immunol.* (2022) 13:954706. 10.3389/fimmu.2022.954706 36032079 PMC9399831

[110] Alpaydın AÖ Turunç TY Avkan-Oğuz V Öner-Eyüboğlu F Tükenmez-Tigen E Hasanoğlu İ Latent tuberculosis infection management in solid organ transplantation recipients: a national snapshot. *Thorac Res Pract.* (2024) 25:130–5. 10.5152/ThoracResPract.2024.23110 39128085 PMC11181162

[111] Yoon SJ Kwon WK Jeong MJ Lee J Oh HY Huh W Comparative evaluation of QuantiFERON-TB Gold Plus for diagnosis of latent tuberculosis infection during solid organ transplantation. *Korean J Transplant.* (2020) 34:8–14. 10.4285/kjt.2020.34.1.8 35770265 PMC9188927

[112] Zeng J Zhu D Zhang H Lin T Song TIGRA-. based INH regimen for prevention of active tuberculosis after kidney transplantation: A single-centre retrospective study. *Int J Antimicrob Agents.* (2024) 63:107093. 10.1016/j.ijantimicag.2024.107093 38244813

[113] Yahav D Gitman MR Margalit I Avni T Leeflang MMG Husain S. Screening for latent tuberculosis infection in solid organ transplant recipients to predict active disease: a systematic review and meta-analysis of diagnostic studies. *Open Forum Infect Dis.* (2023) 10:ofad324. 10.1093/ofid/ofad324 37559757 PMC10407303

[114] Li LS Yang L Zhuang L Ye ZY Zhao WG Gong WP. From immunology to artificial intelligence: revolutionizing latent tuberculosis infection diagnosis with machine learning. *Mil Med Res.* (2023) 10:58. 10.1186/s40779-023-00490-8 38017571 PMC10685516

[115] Luo Y Xue Y Liu W Song H Huang Y Tang G Convolutional neural network based on T-SPOT.TB assay promoting the discrimination between active tuberculosis and latent tuberculosis infection. *Diagn Microbiol Infect Dis.* (2023) 105:115892. 10.1016/j.diagmicrobio.2023.115892 36702072

[116] Abedi S Moosazadeh M Afshari M Charati JY Nezammahalleh A. Determinant factors for mortality during treatment among tuberculosis patients: cox proportional hazards model. *Indian J Tuberc.* (2019) 66:39–43. 10.1016/j.ijtb.2017.05.001 30797281

[117] Wu J Bai J Wang W Xi L Zhang P Lan J ATBdiscrimination: an in silico tool for identification of active tuberculosis disease based on routine blood test and T-SPOT.TB detection results. *J Chem Inf Model.* (2019) 59:4561–8. 10.1021/acs.jcim.9b00678 31609612

[118] Gupta RK Calderwood CJ Yavlinsky A Krutikov M Quartagno M Aichelburg MC Discovery and validation of a personalized risk predictor for incident tuberculosis in low transmission settings. *Nat Med.* (2020) 26:1941–9. 10.1038/s41591-020-1076-0 33077958 PMC7614810

[119] Houben RM Dodd PJ. The global burden of latent tuberculosis infection: a re-estimation using mathematical modelling. *PLoS Med.* (2016) 13:e1002152. 10.1371/journal.pmed.1002152 27780211 PMC5079585

[120] Eremenko EP Borodulina EA Sergeeva IA Kudlay DA Borodulin BE. Recombinant in vitro test T-SPOT.TB as a screening method for early diagnosis of tuberculosis infection. *Tuberc Lung Dis.* (2020) 98:48–52. 10.21292/2075-1230-2020-98-4-48-52

[121] Borodulina EA Kudlay DA Kuznetsova AN. Screening for tuberculosis of patients with HIV-infection, new possibilities. *Acta Biomed Sci.* (2022) 7:83–90.

[122] Adams S Ehrlich R Baatjies R Dendukuri N Wang Z Dheda K. Evaluating latent tuberculosis infection test performance using latent class analysis in a TB and HIV endemic setting. *Int J Environ Res Public Health.* (2019) 16:2912. 10.3390/ijerph16162912 31416206 PMC6720895

[123] Nasiri MJ Pormohammad A Goudarzi H Mardani M Zamani S Migliori GB Latent tuberculosis infection in transplant candidates: a systematic review and meta-analysis on TST and IGRA. *Infection.* (2019) 47:353–61. 10.1007/s15010-019-01285-7 30805899

[124] Wei Z Li Y Wei C Li Y Xu H Wu Y The meta-analysis for ideal cytokines to distinguish the latent and active TB infection. *BMC Pulm Med.* (2020) 20:248. 10.1186/s12890-020-01280-x 32948170 PMC7502022

[125] Peng L Ma W Zhong L Yang J Wu H Zhu L Diagnostic accuracy of *Mycobacterium tuberculosis* antigen-based skin tests (TBSTs) for tuberculosis infection compared with TST and IGRA: a network meta-analysis. *Pathogens.* (2024) 13:1050. 10.3390/pathogens13121050 39770310 PMC11728611

[126] Della Bella C Motisi MA Venturini E D’Elios S Asvestopoulou E Tamborino AM Performance evaluation of the LIOFeron^®^TB/LTBI IGRA for screening of paediatric LTBI and tuberculosis. *Eur J Pediatr* (2025) 184:147. 10.1007/s00431-025-05972-6 39831989 PMC11753305

[127] Aksenova VA Vasilyeva IA Kasaeva TC Samoilova AG Pshenichnaya NY Tyulkova TE. Latent tuberculosis infection in children and adolescents in Russia. *Int J Infect Dis.* (2020) 92:S26–30. 10.1016/j.ijid.2020.02.038 32114196

